# Probing the single pair rupture force of supramolecular quadruply hydrogen bonding modules by nano-adhesion measurement†

**DOI:** 10.1039/c8ra03739f

**Published:** 2018-06-13

**Authors:** Lulu Wang, Zhaoming Yin, Yagang Zhang, Yingfang Jiang, Letao Zhang, Akram Yasin

**Affiliations:** Department of Chemical and Environmental Engineering, Xinjiang Institute of Engineering Urumqi 830023 China ygzhang@ms.xjb.ac.cn +86-991-3838957 +86-18129307169; Xinjiang Technical Institute of Physics and Chemistry, Chinese Academy of Sciences Urumqi 830011 China; University of Chinese Academy of Sciences Beijing 100049 China

## Abstract

Studying quadruply hydrogen bonding (QHB) module interactions in materials matrices presents a significant challenge because a wide variety of non-covalent interactions may be relevant. Here we introduce a method of surface modification with DeUG (7-deazaguanine urea), DAN (2,7-diamido-1,8-naphthyridine) and UPy (2-ureido-4[1*H*]-pyrimidone) modules to form self-assembled monolayers (SAMs) on a glass surface. The QHB interactions under mechanical stress were investigated by measuring adhesion force using PS-DAN (DAN modified polystyrene), PBMA-DeUG (DeUG modified poly butyl methacrylate) and PBA-UPy (UPy modified poly butyl acrylate) as adhesion promoters. A mechanical lap-shear test was used to evaluate the fracture resistance of QHB heterocomplexes. The maximum load at fail showed that QHB interaction contributed significantly (72%) to overall adhesion. For the QHB modified glass surface, using a polymer modified with its complementary QHB partner greatly facilitated their pairing efficiency, up to 40% for DAN-DeUG. A general method from which single pair ruptures force of QHB modules could be obtained using thermodynamic data obtained from solution chemistry was proposed. Using this method, the single pair rupture force for UPy–UPy was measured as 160 pN, and the single pair rupture force for DAN-DeUG was obtained as 193 pN.

## Abstract 2

Quadruply hydrogen bonding interactions under mechanical stress were investigated by measuring adhesion force using PS-DAN, PBMA-DeUG and PBA-UPy as adhesion promoters. Results showed QHB interaction contributed significantly (72%) of overall adhesion.

## Graphical abstract

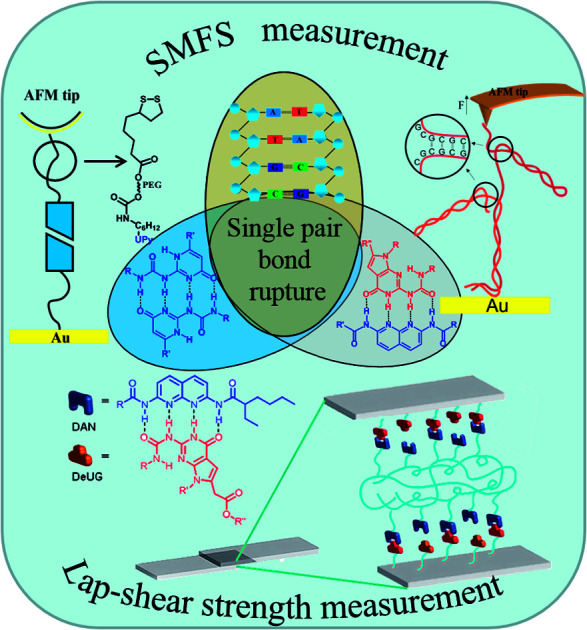

## 1. Introduction

Of the hydrogen bonding interactions which govern the formation of well-defined architectures in supramolecular self-assembly,^1–2^ those that feature quadruply hydrogen bonding (QHB) sites are exceptionally desirable because they usually pair with high affinity and high fidelity.^3^ In particular, the highly stable UPy dimers developed by Meijer and Sijbesma,^4–5^ and the high fidelity DAN·DeAP (deazapterin)^6^ and DAN·UG (butylurea of guanosine)^7^ heterodimers developed by Zimmerman are especially appealing because beyond the stable complexes that they form, they are synthetically accessible. Indeed, several syntheses of the DAN unit are now available^8–12^ as well as optimized DeUG modules bearing synthetic handles for further elaboration.^13^ The binding strength and stability of the UPy dimer^14–15^ and DAN·UG^16^ and DAN·DeUG heterodimers have been investigated.^17–18^ The QHB interactions (such as in DAN-DeUG, DAN-UPy and DAN-UG) are strong (Δ*H* = 25–30 kJ mol^−1^ as compared to C–C bond-dissociation energy ∼350 kJ mol^−1^). These types of interactions offer great potential for smart materials as they are dynamically reversible and responsive to external stimuli such as heat, light, mechanical stress and solvent. Furthermore, materials containing these units could serve as nano-adhesion promoters, energy dissipators and stimuli-responsive polymers^2,4,30^

It has been a long-standing goal to establish structure-property relationship in advanced functional material. Despite the many examples of supramolecular polymers^19^ based on QHB modules^20–21^ and polymers modified with those motifs at side chain,^22^ end chain^23^ and supramolecular ABC triblock polymer,^24^ attempts to reveal how these QHB modules behave in material matrix are remarkably few^25^ especially for a single QHB pair rupture event.^26^ Examples tried to tackle the problem was limited to measuring solution viscosity, surface morphology,^27^ glass transition temperature,^28^ thermo gravimetric analysis and differential scanning calorimetry.^29^ The structure-property relationship of polymers containing QHB modules is far from established. When QHB modules were engineered onto solid surface or into polymer matrix, they lose great amount of degree of freedom in terms of ability to move compared to a small molecule in solution, which would greatly influence their pairing efficiency and response to mechanical stress.

Study QHB interactions in material matrix presents a significant challenge because a wide variety of non-covalent interactions may be relevant.^30^ With great progress made over the years, several very fundamental yet significant questions remain unsolved which could not be evaded from such as (a) how much force does it take to pull apart a single QHB pair? (b) When QHB modules are engineered on solid surface or into polymer matrix, are they still be able to interact like they do in the solution? Also, for a designed responsive behavior, how much contribution is from specific QHB and how much is from non-specific interactions?

Surface modification has become critical means to study non-covalent interactions for complex system.^31,32^ In the work reported here, we demonstrated that with surface modified with QHB modules, measuring adhesion force of paired complexes on sub-nano mole scale was simple, yet effective method to unveil interactions of DAN-DeUG on nano-scale. At molecular level, it was QHB rendered interfacial adhesion which determined the final property of the materials. Mechanical lap-shear test was used to evaluate the rupture event of QHB heterocomplexes. The difference in shear strength (maximum load at fail) was found to be correlated to specific QHB interactions. By comparing shear strength of functionized glass surfaces and polymers to various controls, maximum load at fail showed DAN-DeUG interaction contributed significantly (72%) of overall adhesion due to their pairing on solid surface and in polymer matrix. For DeUG/DAN modified glass surface, using polymer modified with its complementary QHB partner greatly facilitate their pairing efficiency to up to 40%. A general method from which single pair rupture force of QHB modules could be calculated using enthalpy (Δ*H*) was also proposed.

## 2. Materials and methods

### 2.1 Materials

With the exception of 1-(3-dimethylaminopropyl)-3-ethylcarbodiimide hydrochloride (EDC) which was purchased from Advanced ChemTech, dimethylethoxylsilane was purchased from Alfa Aesar and used as received, all other chemicals were purchased from Sigma-Aldrich and used without further purification. Solvents were reagent grade and used without further purification except follows: *N*,*N*-dimethylformamide (DMF) and dimethyl sulfoxide (DMSO) was vacuum distilled from 4 Å molecular sieves just prior to use. Methylene chloride (CH_2_Cl_2_) was obtained from MB-SPS Solvent Purification System and stored under 4 Å molecular sieves prior to use for peptide coupling reaction. For glass slide and silicon wafer surface modification, reagent grade CH_2_Cl_2_ was used. Pyridine was distilled from CaH_2_ and stored under 4 Å molecular sieves. Si wafer and glass slides were purchased from Ted Pella Inc. 10 × 10 mm diced. Si (111) P-type 460–536 μm, 1–30 ohms, no SiO_2_ coating. Gold Seal glass micro slides (soda lime glass) 3 × 1′′, thickness: 0.93–1.05 mm.

### 2.2 Static water contact angle

Static water contact angles were measured using Ramé–Hart contact angle Goniometer with DROP image CA software. 10 μL of distilled water was injected onto the sample surface, allowing the drop to equilibrate for 10 s, and record the mean contact angle (average of left right contact angle). With two modified Si wafers or glass slides of each type, 6 spots for each sample were measured. Error represents plus/minus one standard deviation.

### 2.3 X-ray photoelectron spectroscopy (XPS)

X-ray photoelectron spectra were recorded with a KRATOS AXIS ULTRA XPS system with mono-chromatized Al Kα radiation (1486.6 eV) as the excitation source and a hemispherical analyzer with 165 mm radius. The takeoff angle was set to 90°. The modified glass slides with a size of 1 cm × 1 cm were mounted on sample stubs with carbon tape. Spectra were recorded with a pass energy of 160.0 eV (survey scans) or 40.0 eV (high-resolution scans). Atomic concentrations of elements within the electron escape depth were determined by evaluating the integral intensities of N 1s, F 1s, Si 2p, O 1s and C 1s signals and taking into account the tabulated atomic sensitivity factors and the instrument transmission.^33^ The spectra were referenced by setting the peaks of the saturated hydrocarbon C 1s to 285.0 eV. The pressure in the analytical chamber during analysis was approximately 2 × 10^−9^ torr. Spectra were recorded with a 200 μm in diameter spot size.

### 2.4 Atomic force microscopy (AFM)

Surface roughness was measured using Asylum Research MFP-3D™ with IGOR Pro software. Tapping mode at scan rate 1.0 Hz with 512 lines by 512 points was carried out on 3 μm × 3 μm area using Tap 300 Al AFM probe from Budget Sensors (tip radius < 10 nm, rotated monolithic silicon probe, resonant freq. 300 kHz(±100 kHz), force constant 40 N m^−1^ (range 20–75 N m^−1^), symmetric tip shape, chip size 3.4 × 1.6 × 0.3 mm, 30 nm Al for enhanced reflectivity). A first order flattening routine was applied prior to calculation of the surface roughness. Root-mean-squared (RMS) roughness was calculated using data on 3 μm × 3 μm scan region.

### 2.5 Ellipsometry

Film thickness of modified silicon wafers was measured using J. A. Woollam Co. variable-angle spectroscopic ellipsometer. Ellipsometric data were acquired *via* spectroscopic scan with angle of incidence at 50, 60 and 70° and spectral range: 300–1000 nm with revolutions per measurement (Revs/Meas) set at 10. Spot sized analyzed was 1 mm in diameter when incident light is normal to the surface, will be larger when scan with angle of incidence at 50, 60 and 70°. Measurements were made 7 times for each type of sample. The data was fitted *via* layer by layer model according to the manual using WVASE 32™ software. Error represents plus/minus one standard deviation.

### 2.6 Nuclear magnetic resonance (NMR) and mass spectroscopy (MS)

^1^H NMR spectra were acquired using Varian Unity 500 MHz (^13^C, 126 MHz) spectrometer. ^1^H NMR chemical shifts (*δ*) are reported in parts per million (ppm) and were referenced to the residual solvent peak at 7.26 ppm for CDCl_3_ and 2.50 ppm for DMSO-*d*_6_. ^13^C NMR chemical shifts are reported in ppm and were referenced to the residual solvent peak at 77.16 ppm for CDCl_3_ and 39.52 ppm for DMSO-*d*_6_. All NMR spectra were original one which was scanned. Mass spectra were obtained on Micromass Q-Tof Ultima (HR-ESI) and Micromass Quattro (LR-ESI) instruments. MALDI-TOF-MS was carried out using Applied Biosystems Voyager-DE STR with a nitrogen laser (337 nm, 3 ns pulse, 20 Hz maximum firing rate) and using 2-(4′-hydroxybenzeneazo)benzoic acid (HABA) as matrix.

### 2.7 Adhesion measurements *via* lap-shear experiment

Adhesion was measured using Instron Mini 44 load-frame equipped with Labview 5.1 software. Each lap-shear sample was prepared as following: A pair of glass slides was set using 10 μL of 10 mg mL^−1^ of each polymer solution in CH_2_Cl_2_ with contact area 1.5 cm × 2.5 cm. The sample was clamped with binder clips and cured at room temperature for 24 h before lap shear test. Crosshead speed limit is set at 1.0 mm min^−1^ Load (kg) *vs.* position was plotted and maximum load at fail was recorded. Each data set contains 10 measures. Multiplying the average maximum load at fail by gravitational acceleration constant and divided by contact area give the shear strength in MPa. Error represents plus/minus one standard deviation.

## 3. Results and discussion

### 3.1 Synthesis of silane coupled UPy, DeUG and DAN monomers

**Fig. 1:**
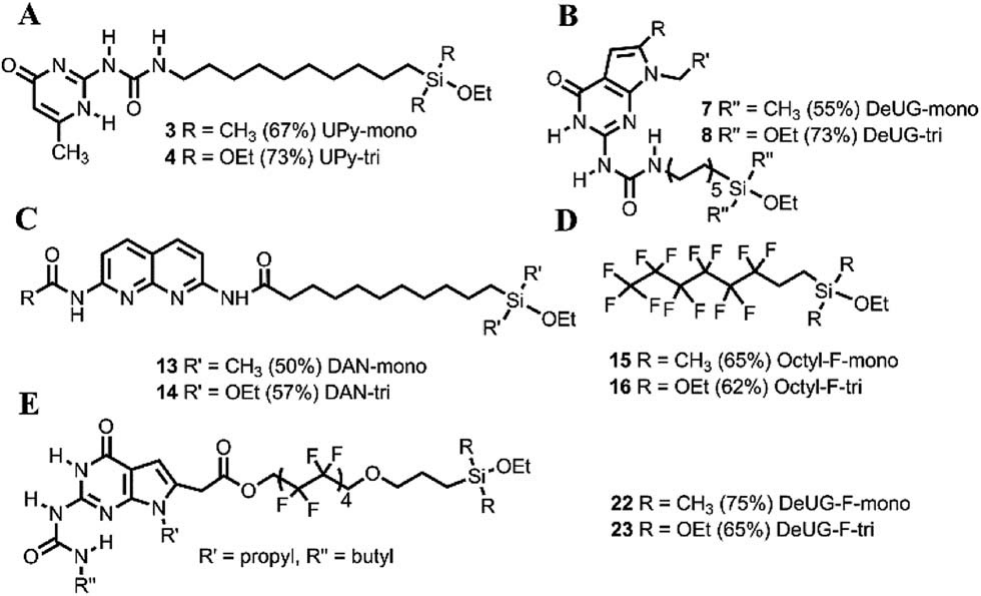
Structure of QHB monomers (A) silane coupled UPy, (B) silane coupled DeUG, (C) silane coupled DAN, (D) silane coupled fluorinated carbon chain monomer, (E) silane coupled DeUG monomers with fluorinated carbon chain linker.

The QHB monomers were synthesized by coupling reactions, as depicted in Fig. 1. Detailed routes of synthesis were presented in ESI Scheme S1 and S2.† Silane coupled UPy monomers were obtained *via* hydrosilylation of UPy precursor afforded monoethoxylsilane monomer 3 in 67% yield and triethoxylsilane monomer 4 in 73% yield (Fig. 1A). DeUG precursor was treated with 10-isocyanatodec-1-ene afforded DeUG with terminal alkenes carbon chain in 60% yield. Hydrosilylation of DeUG precursor afforded DeUG coupled monoethoxylsilane monomer 7 in 55% yield and triethoxylsilane monomer 8 in 73% yield (Fig. 1B). Bromination and reductive amination of DAN afforded DAN precursor in 67% yield. The coupling of undec-10-enoic acid with DAN precursor was achieved using peptide coupling method.^34^ Hydrosilylation of terminal alkenes afforded DAN precursor coupled monoethoxylsilane monomer 13 in 50% yield and triethoxylsilane monomer 14 in 57% yield (Fig. 1C).

Hydrosilylation of 3,3,4,4,5,5,6,6,7,7,8,8,8-tridecafluoro-1-octene afforded corresponding monoethoxyl silane 15 in 65% yield and triethoxylmonomer 16 in 62% yield (Fig. 1D). Coupling DAN unit ^20^ with allylic substitution fluorinated carbon chain linker was achieved *via* Steglich esterification^35,36^ and hydrosilylation of afforded DeUG precursor coupled monoethoxylsilane monomer 22 in 75% yield and triethoxylsilane monomer 23 in 65% yield (Fig. 1E). Both 22 and 23 has fluorinated linker between QHB motifs and silane anchoring site.^18^ Detailed procedure see Fig. S1 and S2.†

### 3.2 Modification and characterization of glass slides and Si wafer surfaces

**Fig. 2:**
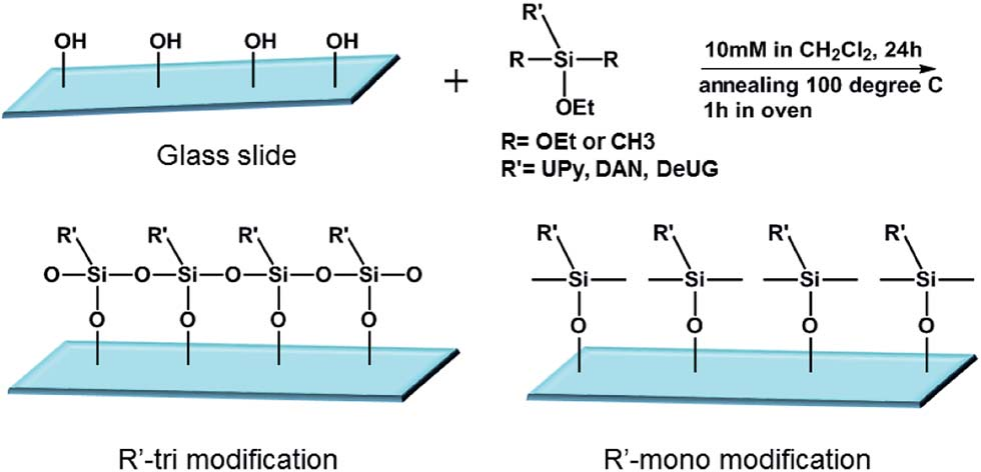
Suface modification of glass slides with QHB monomers.

Surface modification of glass slides and Si wafers with QHB monomers. Si wafers/glass slides were cleaned in Piranha (concentrated H_2_SO_4_: 30% H_2_O_2_, 3 : 1 v/v) at 100 °C for 1 h. Immediately following the cleaning, silicon wafers/glass slides were rinsed thoroughly with Millipore water, then anhydrous ethanol, and were treated under a stream of dry nitrogen. Freshly cleaned silicon wafers/glass slides were immersed in 10 mM specific silane monomer solution or a mixture of two monomers (1 : 1 mole ratio) (for mixed SAMs) solution in CH_2_Cl_2_ at room temperature for 24 h, Si wafers/glass slides were taken out form the solution, rinsed with CH_2_Cl_2_ and annealed at 100 °C for 1 h, cooled to room temperature and rinsed thoroughly with dichloromethane, acetone, Millipore water, then anhydrous ethanol, and were dried under a stream of dry nitrogen (Fig. 2).

**Fig. 3:**
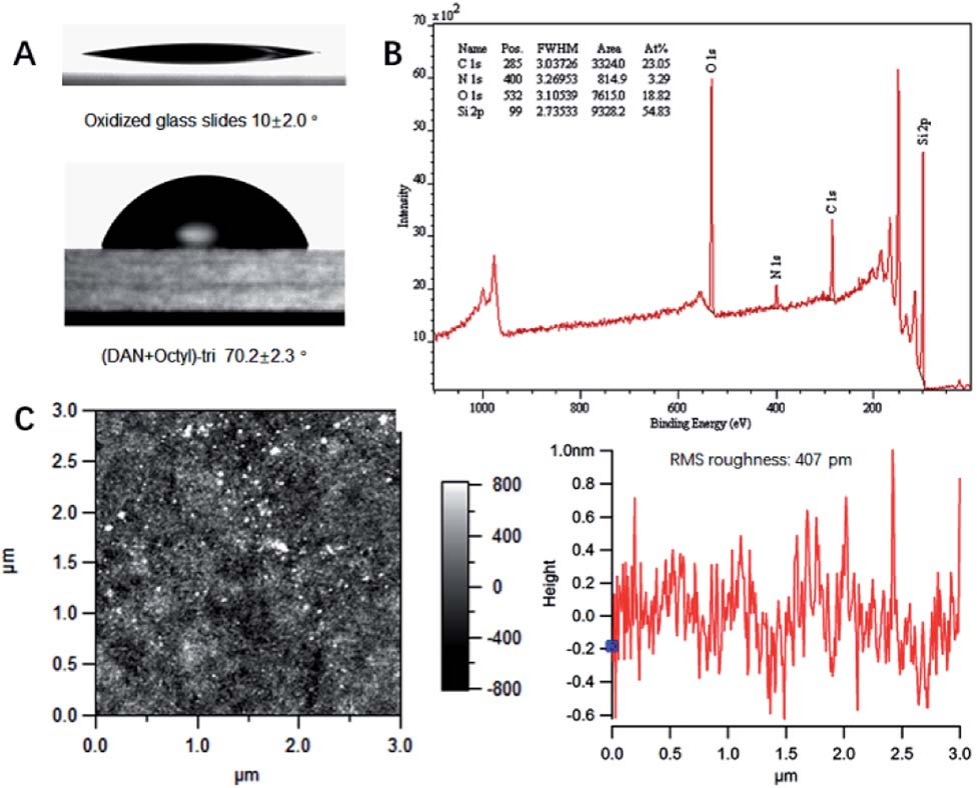
(A) Static water contact angle of modified glass slides with (DAN + octyl)-tri; (B) XPS survey spectra of modified glass slides with (DAN + octyl)-tri; (C) AFM height image of glass slides modified with (DeUG + octyl)-tri and section graph of AFM height image of glass slides modified with (DeUG + octyl)-tri with root mean square.

SAMs on glass slides and Si wafers were prepared using various silane monomers bearing UPy, DeUG and DAN modules with either alkyl chain or fluorinated alkyl chain linker. Mixed SAMs were also synthesized using 1 : 1 mole ratio of QHB coupled silane monomer and alkyl/fluorinated alkyl silane monomer. Modified surface was characterized with various techniques to validate the effective of silane monomer deposition (see ESI†). Static water contact angle of unmodified/modified surfaces correlated well with relative polarity of corresponding functional groups. For example, compared to Piranha treated glass slides (contact angle 10°), (DAN + octyl)-tri modified glass slides surface has contact angle 70.2° (see ESI, Table S1 and Fig. S20 and S21†). Survey spectra of X-ray photoelectron spectroscopy (XPS) showed characteristic peaks belong to specific element. High resolution XPS scans of carbon regions of modified glass slides revealed alkyl carbon, O and N bonded carbon at specific binding energy (Fig. 3B); Atomic force microscopy (AFM) height image of glass slides modified with (DeUG + octyl)-tri and section graph of modified glass slides with (DeUG + octyl)-tri with root mean square roughness = 407 pm (Fig. 3C). Atomic composition from XPS survey spectra implied surface modified with mono-alkoxyl silane monomer has low surface loading of QHB modules as compared to tri-alkoxyl silane modified surface. The results were consistent with the fact that trialkoxyl silane could condense adjacently thus form more densely packed SAMs. Atomic force microscopy (AFM) height image and section graph of modified glass slides demonstrated relatively uniform surface with root mean square roughness < 500 pm. Matrix-assisted laser desorption time-of-flight mass spectrometer (MALDI-TOF-MS) provided extra evidence of the success of surface modification by identified fragments generated from corresponding QHB modules (see ESI, Fig. S22 and S23†).

Ellipsometric data were acquired *via* spectroscopic scan with angle of incidence at 50, 60 and 70° and spectral range: 300–1000 nm with revolutions per measurement (Revs/Meas) set at 10. Spot sized analyzed was 1 mm in diameter when incident light is normal to the surface, will be larger when scan with angle of incidence at 50, 60 and 70°. For Piranha treated Si wafer, sequentially add Si, SiO_2_ layer, fix Si layer at 1.00 mm, then do a normal fit to obtain thickness of SiO_2_ layer (2.23 nm). For surface modified with various silane monomers, sequentially add Si, SiO_2_, Cauchy layer, fix Si layer at 1.00 mm, SiO_2_ layer at 2.23 nm, and then do a normal fit to obtain thickness of SAM layer. For example, the thickness of SAMs DeUG-triethoxyl silane modified Si wafer surface was measured by ellipsometry. Thickness of the SAM layer 1.971 nm, calculated MSE 1.495. The thickness of film measured on modified surface is consistent with SAMs as compared to the theoretical thickness (see ESI, Table S2†).

### 3.3 Lap-shear experiment to measure adhesion between modified glass surfaces using QHB modified polymers

**Fig. 4:**
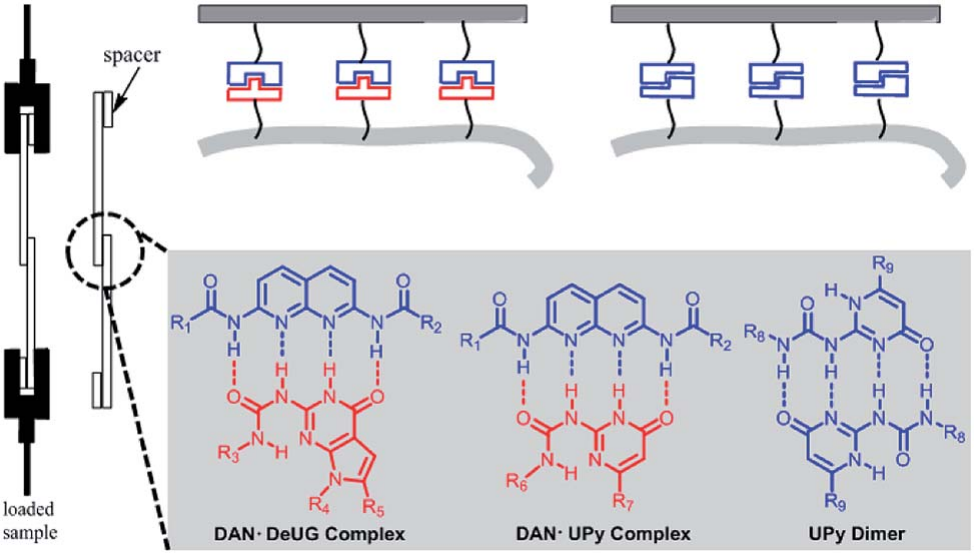
Three types of QHB complex: DAN-DeUG, DAN-UPy and UPy dimer focused in lap-shear measurements.

Focused here are three types of QHB complex: DAN-DeUG, DAN-UPy and UPy dimer (Fig. 4). The effectiveness of their use as adhesion promoters and their pairing efficiency were systematically investigated. After pioneering work of Nuzzo^37^ and Whitesides,^38^ SAMs on materials surface has been routinely reported. One widely used method was stepwise modification of hydroxyl rich surface with silane monomers such as aminopropytriethoxylsilane. The intrinsic limitation was that unreacted functional groups and linkages formed in each step generating non-specific interactions. Here we aim to develop strategy for synthesizing QHB coupled silane monomers which allows one-step surface modification to form SAMs. Trialkoxylsilane may form polymeric siloxane type structure which could lead to non-uniformed surface.^39^ It was reported that fluorocarbon chains could self-organize and lead to well packed SAMs^40^ and mixed SAMs could potentially increase the accessibility of targeted functional groups.^41^ We set out to synthesize QHB modules coupled silane monomers (both triethoxyl and monoethoxyl) with alkyl linker or fluorinated carbon chain linker and use them for glass surface modification to probe single pair rupture force of QHB modules. The resulting modified surfaces proved to be an ideal system to study the behavior of QHB modules on by adhesion measurement.

**Fig. 5:**
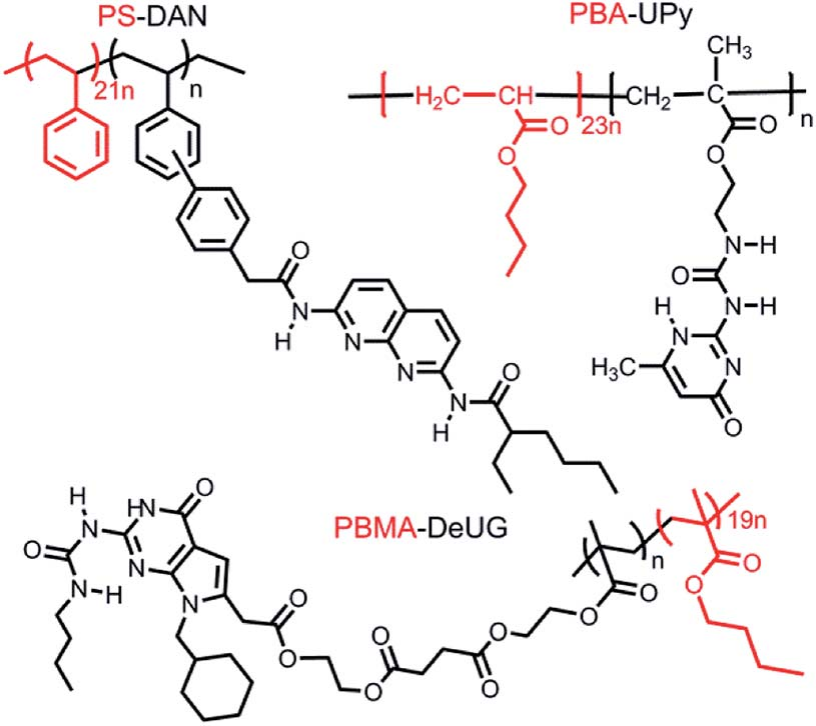
Three QHB module modified polymers as adhesion promoters.

**Fig. 6:**
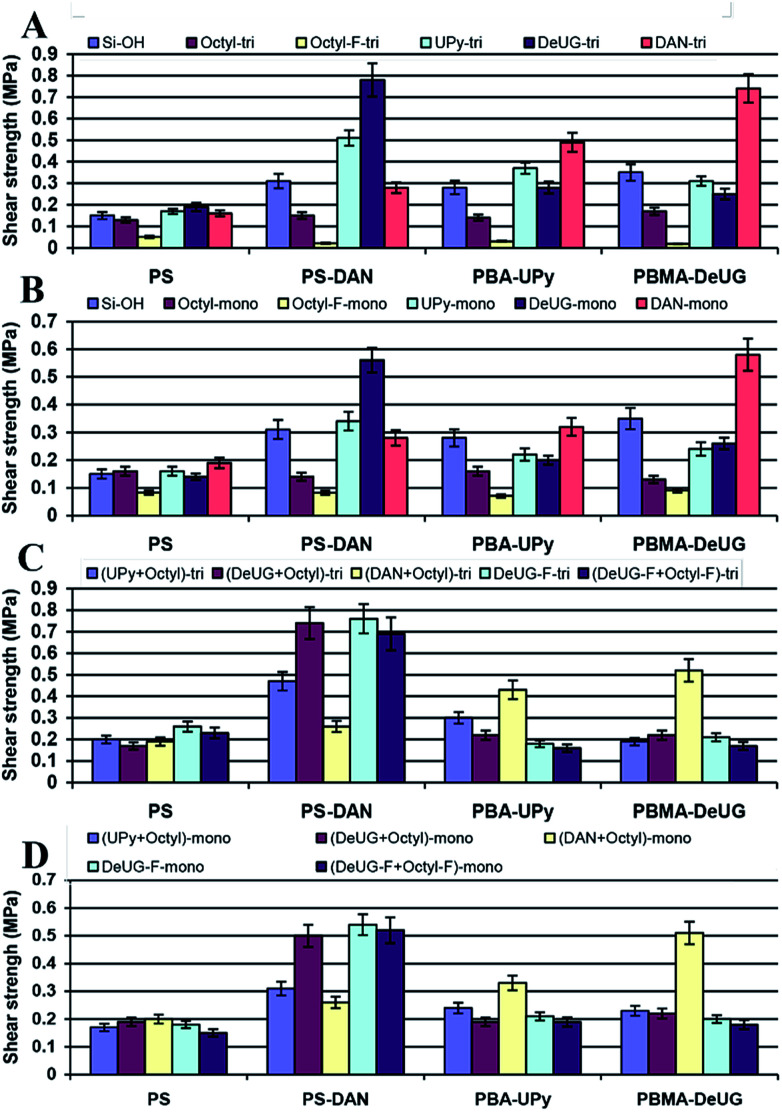
Lap-shear strength measurement for glass slides surfaces modified with QHB modules polymers (A) triethoxylsilane monomers, (B) monoethoxylsilane monomers, (C) triethoxylsilane monomers (mixed SAMs). (D) Monoethoxylsilane monomers (mixed SAMs).

No adhesion was observed directly between two glass slides/Si wafer surfaces modified with complimentary QHB modules described in Fig. 6. This was probably due to very low pairing efficiency considering the fact that once QHB modules were fixed onto solid surface, they lose great amount of degree of freedom in terms of ability to move and pair. In order to improve the pairing efficiency, PS-DAN, PBMA-DeUG and PBA-UPy were designed (Fig. 5). Three types of polymers: PS-DAN (4.5 mol%, *M*_n_ = 73 KDa, PDI = 1.8), PBMA-DeUG (5.0 mol%, *M*_n_ = 18.5 KDa, PDI = 1.2) and PBA-UPy (4.1 mol%, *M*_n_ = 38 KDa, PDI = 2.1) were synthesized and used as adhesion promoters for modified glass slide surface. PS (*M*_n_ = 69 KDa, PDI = 2.0) was used as control polymer.

In lap-shear experiment, a pair of glass slides was set using 10 μL of 10 mg mL^−1^ of each polymer solution in CH_2_Cl_2_ with contact area 1.5 cm × 2.5 cm. The sample was clamped with binder clips and cured at room temperature for 24 h before lap shear test. Load *versus* position was plotted and maximum load at fail was recorded and converted to shear strength in MPa. 10 μL of 10 mg mL^−1^ of polymer solution was proved to be optimal as adhesion promoters (see ESI, Fig. S24 and S25†). These polymer solutions were used as adhesion promoters for modified glass slide surface and showed greatly enhanced adhesion. Shear strength using different polymer as adhesion promoters for various QHB module modified glass slides were shown in Fig. 6. Lap-shear experiment using Si modified with UPy, DeUG and DAN modules was not successful due to its brittle nature and small size of the Si wafer (10 × 10 mm).

There were good correlation between shear strength and QHB specificity as each QHB modified polymer demonstrated greatest shear strength towards the surface modified with its complimentary QHB partner. This was solid evidence that these QHB modules could pair and be effective adhesion promoters under appropriate conditions. The shear strength was relatively lower for surface modified with monoalkoxyl silane monomers as compared to trialkoxyl silane monomer (Fig. 6A and B). Mixed SAMs seemed to improve the accessibility of QHB modules on surface based on the fact that they showed similar shear strength compared to normal SAMs (2^nd^ column set of Fig. 6C and D) while theoretically they only have half amount of QHB modules on the same surface area. Results showed that surface modified with monomers bearing alkyl linker and fluorinated carbon chain linker behaves similarly as they had close shear strength (Fig. 6C and D). While surface modified with octyl-F-tri demonstrated very low adhesion for QHB module modified polymers (Fig. 6A).

### 3.4 Calculation the single pair rupture force of QHB

Visual inspection under microscope revealed a mixed failure mode of adhesive and cohesive failure. The binding strength (Δ*H*) of QHB pairs fell in the range of 25–30 KJ mol^−1^ compared to C–C bond dissociation energy which is ∼350 KJ mol^−1^. Theoretically the fractured surface tends to yield adhesive failure mode, however considering loaded stress is distributed unevenly in lap-shear rupture process,^42^ the mixed failure mode of adhesive and cohesive was actually quite reasonable.

Lap-Shear experiment was used to investigate the quardruple hydrogen bonding pair interaction at molecular level and to calculate single pair rupture force. Gaub *et al.* experimentally showed that the unbinding forces of avidin-biotin complex are proportional to the enthalpy change of the complex formation but independent of changes in the free energy and entropy.^43^ Their results indicated that unbinding process was adiabatic and entropic changes occurred after unbinding. Molecular mechanics simulation of streptavidin–biotin interactions also indicated that rupture strength correlates with enthalpies rather than free energies.^44^

Along these lines, we proposed here a general method using enthalpy associated with QHB hetero-complex to calculate single pair rupture force of QHB modules using eqn (1).

1 *W* = *F* × *S* = Δ*H* × *N*_A_

where *W* is the mechanical work needed to break the hydrogen bonding pair, *F* is the force needed, *S* is the distance of the applied force, Δ*H* is enthalpy, *N*_A_ is the Avogadro constant, Δ*H* is enthalpy, which can be measured using solution chemistry such as NMR titration, ITC and solution viscosity. W can be calculated using Δ*H*.

For single pair rupture force of DeUG-DAN, it was reported that for DeUG-DAN pair in chloroform at 25 °C (298.2 K), *K*_a_ = 1.9 × 10^8^ M^−1^, Δ*H* = −6.9 Kal mol^−1^ (−28980 J mol^−1^) and *T*Δ*S* = 2.8 Kal mol^−1^.^13^ Crystal structure showed H-bonding length of QHBM was around 0.19–0.20 nm.^13^ It was estimated pulling apart 0.25 nm would break the DeUG-DAN complex.^45^ Using eqn (1), the calculated value of DeUG-DAN single pair rupture force was 193 pN.

For UPy dimer formation, it was reported^4,28^ that at 25 °C (298.2 K), *K*_a_ = 10^7^ and at 80 °C (353.2 K), *K*_a_ = 2, respectively. According to Δ*G* = Δ*H* − *T*Δ*S* = −*RT* ln *K*_a_, Δ*H* was calculated as −24 559 J mol^−1^ (−5.89 Kal mol^−1^). It was known typical hydrogen bonding distance was within 0.1–0.2 nm.^45^ It was reported that for UPy dimer, H-bonding distance was within 0.27–0.32 nm.^45^ Assuming pulling apart 0.25 nm would break QHB pair,^46^ single pair rupture force was obtained as 160 pN for UPy dimer (see ESI, S28†) using eqn (1), which was consistent with the experimental value (145 pN) measured by Vancso *et al.*^26^

The stress *versus* extension curve of lap-shear experiment vividly described the dynamic nature of rupture event. The integration area under the rupture curve was used to calculate the amount of adhesion energy/mechanical work involved in the rupture process.^47,48^ Controls were used to approximate non-specific interactions such as London dispersion and dipole–dipole. Subtracting the energy associated with these non-specific interactions from the overall adhesion, the percentage contribution of specific QHB interaction could be calculated. Adhesion energy/mechanical work was calculated using difference between maximum load at fail and plastic deformation load multiply rupture length, divided by two. The apparent rupture length *x* of DAN-DeUG pairs can be calculated using eqn (2).

2 *C* × *N*_A_ × *F* × *x* = *W*

where *C* is the amount of QHB modules (specific adhesion) on glass surface within the lap-shear contact area; *N*_A_ is the Avogadro constant; *F* is the calculated single pair rupture force; *x* is the apparent rupture length of DAN-DeUG pairs; *W* is calculated adhesion energy due to specific DAN-DeUG interaction. The data set of triethoxyl silane monomers modified surface was chosen for the calculation for the following reasons: (1) it had better surface coverage (2) it showed the strongest adhesion when using QHB modules modified polymer as adhesion promoters (3) from practical application perspective, it was the most close to the of QHB modified polymer systems.

The full coverage of perfect SAMs would have 0.83 nmol cm^−2^*via* silane deposition.^49–51^ Technical data of Goldseal glass Microslides from Ted Pella Inc. showed that silicon dioxide is 72% of its all chemical composition. Thus the surface coverage could be estimated as 0.60 nmol cm^−2^. With contact area of lap-shear sample 1.5 cm × 2.5 cm, the amount of QHB modules on each side of glass slides could be calculated as 2.25 nmol. Each lap-shear sample had two surfaces associated, thus the amount of QHB modules would be 4.50 nmol. The apparent rupture length *x* of DAN-DeUG pairs was calculated as 180 nm using eqn (2). It was found that *x* was greater than QHB distance and less than rupture length/stressed extension in lap-shear (0.25 nm ≫ *x* ≪ 0.92 mm). This implied that stretching the polymer chain and stretching the QHB pairs occurred at same time over a quite long stretching process. Rupture length *x* was defined as difference between position at fail and ending point of plastic deformation of glass slides.

**Fig. 7:**
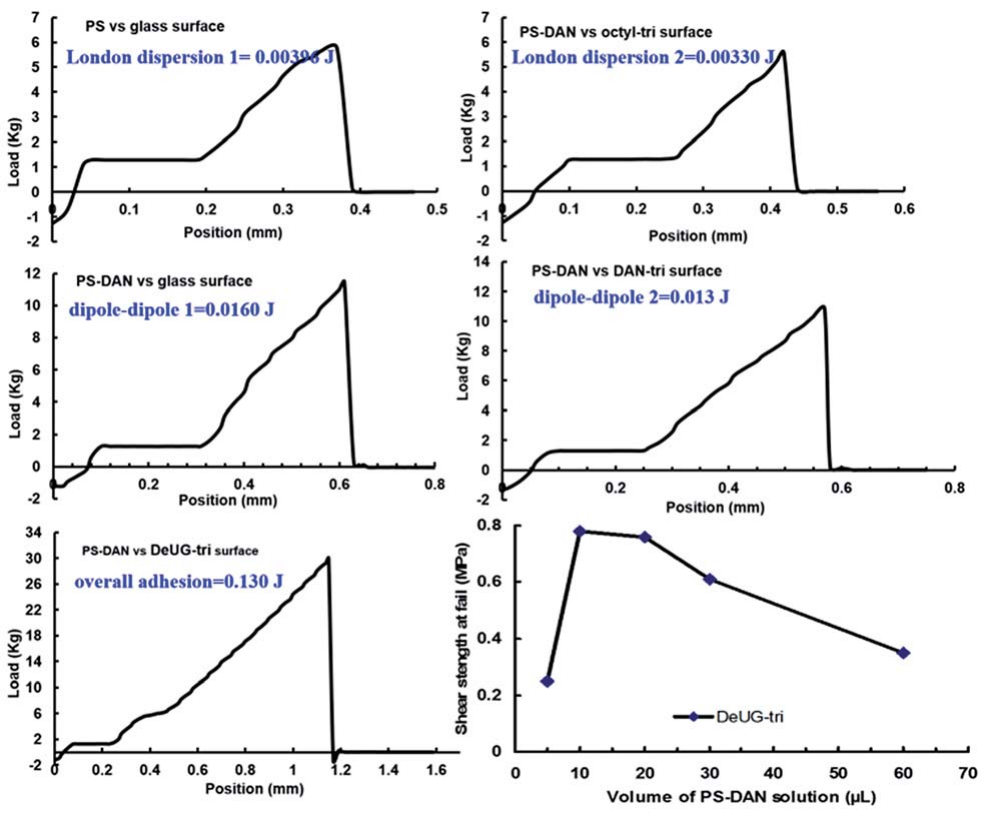
Lap-shear measurement using PS-DAN toward DeUG-tri modified glass surface: PS toward glass surface, adhesion is defined as London dispersion 1; PS-DAN toward octyl-tri modified glass surface, adhesion is defined as London dispersion 2; PS-DAN toward glass surface, adhesion is defined as dipole–dipole 1; PS-DAN toward DAN-tri modified glass surface, adhesion is defined as dipole–dipole 2; PS-DAN toward DeUG-tri modified glass surface, adhesion is defined as overall adhesion. Lap-shear strength at fail with different amount of polymer used was tested using PS-DAN toward DeUG modified surface.

Overall adhesion using PS-DAN toward DeUG-tri modified glass surface could be calculated. The adhesion includes specific DAN-DeUG interaction and non-specific interactions (0.130 J for DAN-DeUG lap-shear), adhesion due to specific QHB interaction can be calculated as 0.094 J for DAN-DeUG in lap-shear (specific adhesion = overall adhesion − non-specific adhesion = overall adhesion − non-(London dispersion 1) − (London dispersion 2) − (dipole–dipole 1) − (dipole–dipole 2) = (0.130 − 0.004 − 0.003 − 0.016 − 0.013)*J* = 0.094*J*, Fig. 7). The amount of QHB modules in PS-DAN, PBMA-DeUG and PBA-UPy was calculated based on *M*_n_ of modified polymers and loading percentage of QHBM. Detailed calculation of process was presented in ESI information S31 and S32.† Quantitatively there were 32–37 nmol QHB modules in the amount of polymer used for each lap-shear setting which was 7–8 equivalents to the surface coverage of QHBM modified glass surface (0.6 nmol cm^−2^, 4.50 nmol in total for two slides with contact area of 1.5 cm × 2.5 cm). Excessive amount of QHB modules in polymer chains greatly promoted the pairing efficiency, but it would also form non-specific interactions which should be subtracted when calculating percentage contribution of specific QHBM in overall adhesion. The percentage contribution of DAN-DeUG interaction was calculated as 72% (see ESI, S31 and S32†) in overall adhesion.

Theoretical adhesion force *F*_T_ for each lap-shear sample can be calculated using eqn (3).

3 *F*_T_ × *x* = Δ*H* × *C*

where *x* is the apparent rupture length; Δ*H* is the enthalpy associated with DAN-DeUG association; *C* is the amount of QHB modules on glass surface within the lap-shear contact area. Considering the soft nature of H-bonding interaction and those QHB pairs were strained over quite long period of distance (sub mm scale), using apparent rupture length (180 nm) should be a close approximation. Theoretical adhesion force *F*_T_ for DAN-DeUG associated lap-shear sample was calculated as 73.9 kg by eqn (3) (experimental observed average maximum load at fail 29.8 kg). Thus the pairing efficiency of DAN-DeUG was calculated as 40.3% (see ESI†). The pairing efficiency of UPy–UPy was about half of the theoretical pairing efficiency of DAN-DeUG, which was reasonable considering UPy could dimerize by itself in polymer chains.

**Fig. 8:**
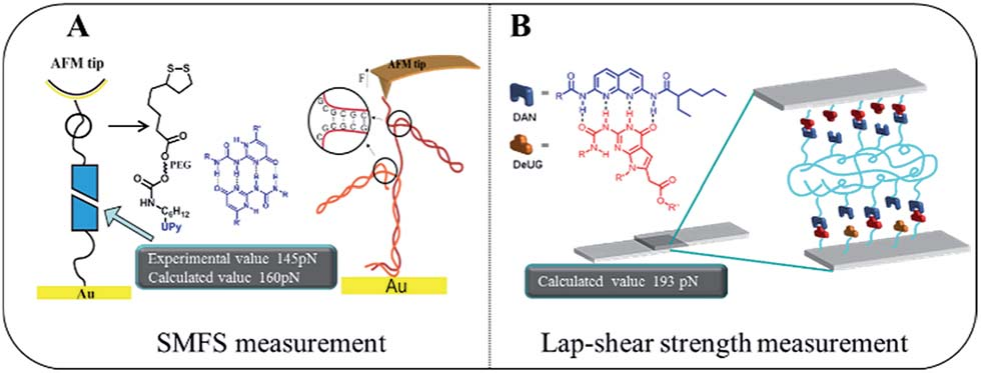
Comparison of microcosmic AFM measurement (A) and macroscopic lap-shear (B) for single pair bond rupture force of QHB UPy–UPy and DAN-DeUG.

Gaub^43^ and Vancso^26^ are two pioneers who set out to probe single pair rupture force of multiple hydrogen bonding include DNA pairs and QHB modules using AFM from microscopic perspective (Fig. 8A). Gaub's group used SMFS to measure sequence-dependent mechanical properties of single DNA and the base-pairing forces of G–C and A–T nucleotides. The force was measured through stretching individual DNA double strands attached between a gold surface and AFM tip. Vancso *et al.* also studied the rupture force of quadruple H-bonded UPy system. In their work, they investigated the QHB single-molecule rupture force with PEG-based telechelic bis(UPy) materials immobilized on Au (111) and AFM tips which was functionalized with pyrimidinone disulfide. In both cases, sophisticate experimental set up such as “fishing” or a peculiar data processing like superposition are required. Interestingly, using the approach proposed here by a macroscopic lap-shear experiment, single pair rupture force for UPy–UPy was measured as 160 pN, and single pair rupture force for DAN-DeUG was obtained as 193 pN. These results were consistent with the experimental value (145 pN) measured by Vancso.^26^ Furthermore, our results demonstrated that QHB modules anchored on materials surface could pair and act as effective adhesion promoters under appropriate conditions (Fig. 8B).

## 4. Conclusions

In summary, we proposed an approach for probing single pair rupture force of supramolecular quadruply hydrogen bonding modules by nano-adhesion measurement. This was achieved by surface modification of glass microscope slides using specifically designed UPy, DeUG and DAN modules. Based on the lap-shear experiment along with data measured from solution studies, a general model and calculation method was established. With this approach, not only one could extract the non-specific interactions from over all surface adhesion, single pair rupture force and pairing efficiency of QHB could also be obtained. Specifically, using this method, single pair rupture force for UPy–UPy was calculated as 160 pN, and single pair rupture force for DAN-DeUG was obtained as 193 pN. Results implied that QHB DAN-DeUG interaction contributed 72% of overall adhesion with a pairing efficiency of 40%. This approach could facilitate better understanding of the recognition process of QHB modules on material surface and interface.

## Conflicts of interest

There are no conflicts to declare.

## Supplementary Material

RA-008-C8RA03739F-s001
