# Supplementary material for: Probing the single pair rupture force of supramolecular quadruply hydrogen bonding modules by nano-adhesion measurement
Source: RSC Adv. 2018 Jun 13;8(39):21798–805. doi: 10.1039/c8ra03739f (PMC9081402; doi:10.1039/c8ra03739f)
Supplement: RA-008-C8RA03739F-s001 [file RA-008-C8RA03739F-s001.pdf]

Electronic Supplementary Material (ESI) for RSC Advances.
This journal is © The Royal Society of Chemistry 2018

Supplementary Information

Probing single pair rupture force of supramolecular quadruply hydrogen bonding modules by nano-adhesion measurement

Lulu Wang,[†,‡,§] *Zhaoming Yin,*[†] *Yagang Zhang ,*[* †,‡,§] *, Yingfang Jiang,*[‡,§] *Letao Zhang,*[‡] *Akram Yasin*[‡]

† Department of Chemical and Environmental Engineering, Xinjiang Institute of Engineering, Urumqi 830023, China

‡ Xinjiang Technical Institute of Physics and Chemistry, the Chinese Academy of Sciences, Urumqi 830011, China

§ University of the Chinese Academy of Sciences, Beijing 100049, China

Corresponding author: Prof. Yagang Zhang

Tel: +86-18129307169; Fax: +86-991-3838957; E-mail: ygzhang@ms.xjb.ac.cn

Table of Contents

1. Synthetic procedure 5
2. ^{1}H and ^{13}C NMR Spectra..11
3. Elemental composition report of HR-ESI-MS 17
4. Static water contact angle measurement……………………………………………………21
5. MALDI-TOF-MS of modified glass slides…………………………………………………24
6. Thickness of mono-layer using ellipsometry measurement 25
7. Adhesion measurements via Lap-shear experiment 26
8. Method to calculate single pair rupture force of QHB modules 28
9. Calculation of percentage contribution of QHB modules in overall adhesion..........................31
10. Pairing efficiency of QHB modules in Lap-shear……………………………………….…32
Reference: 33

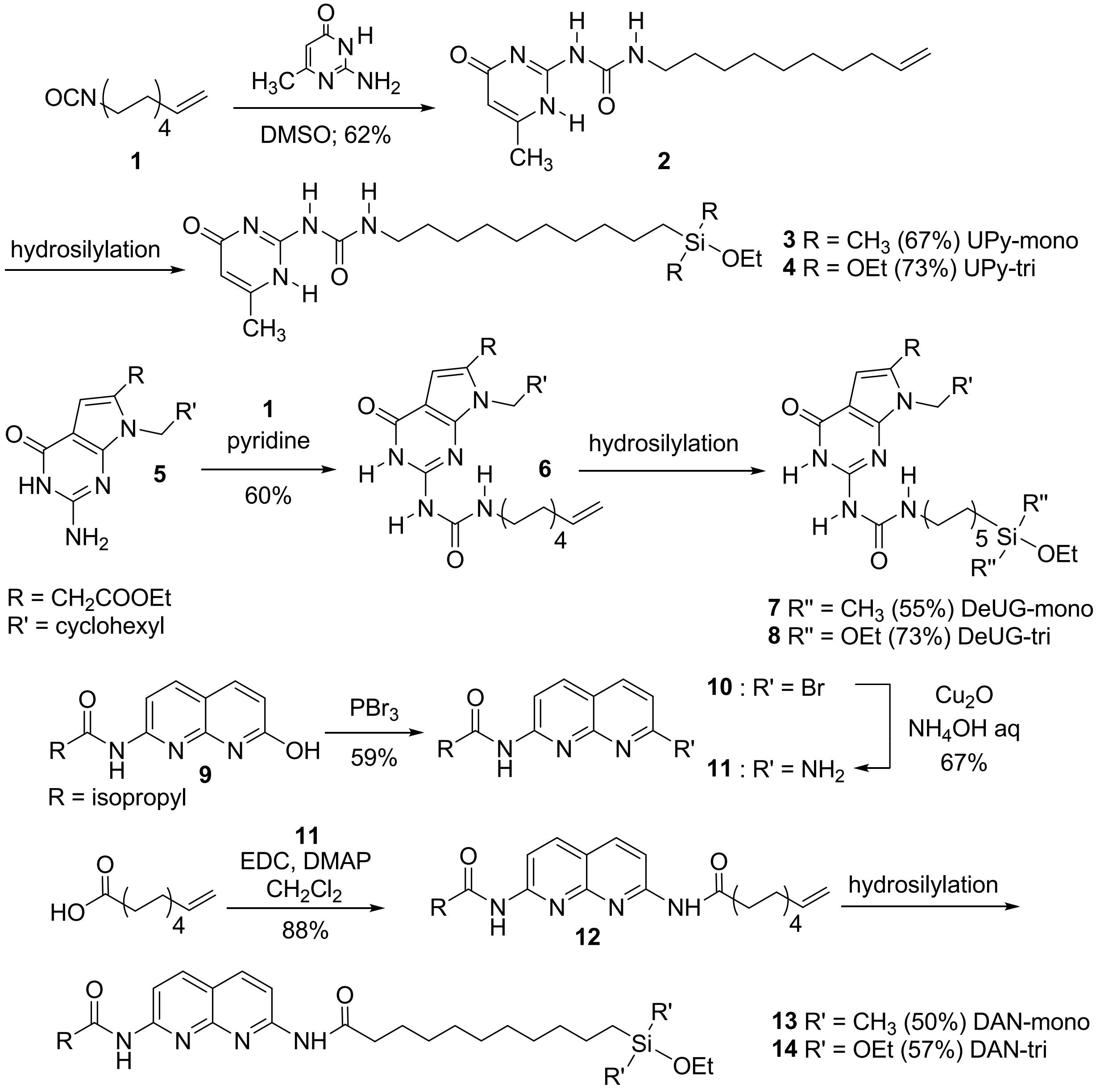

Scheme S1. Synthesis of silane coupled UPy, DeUG and DAN monomers with decyl linker.

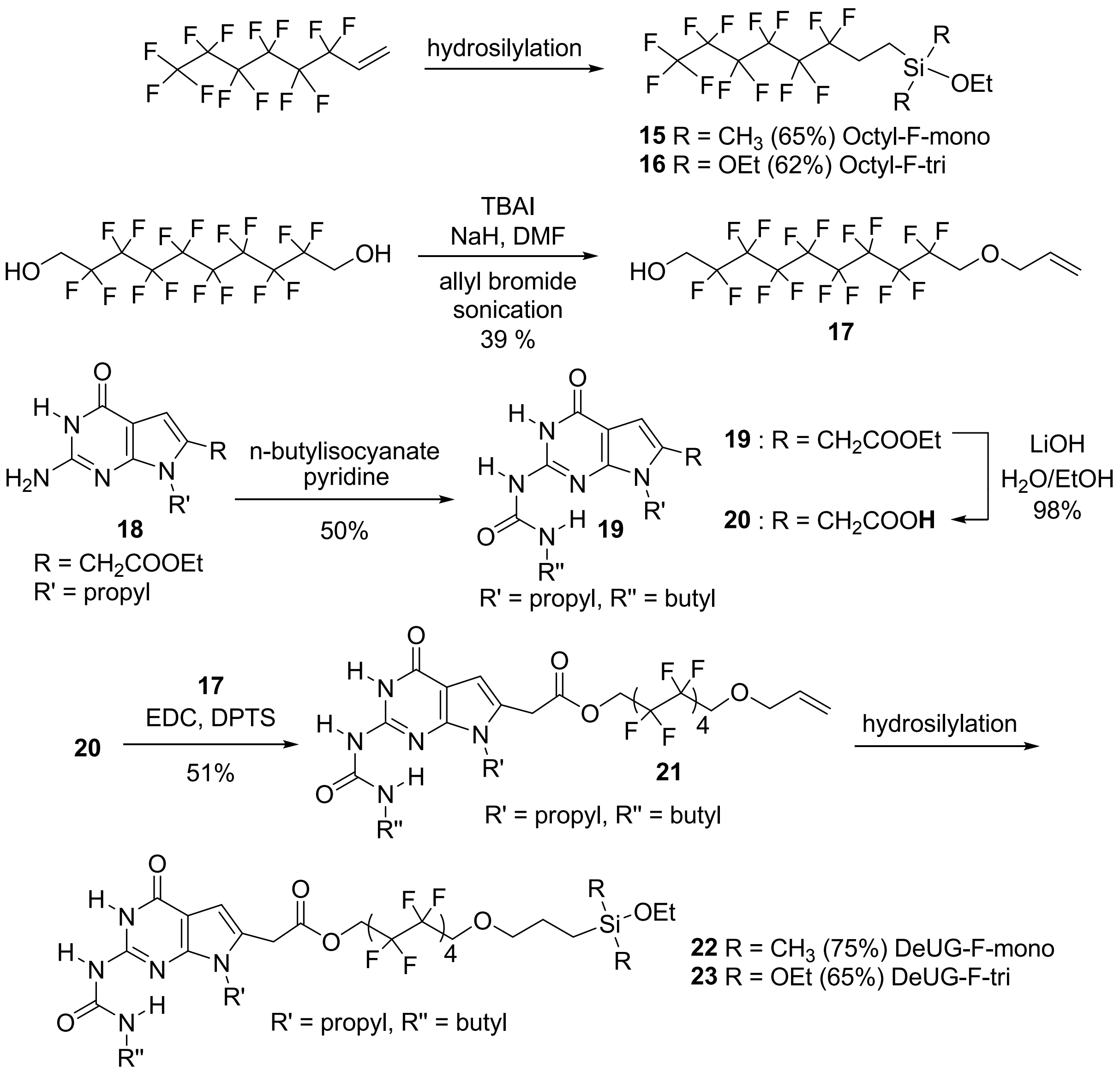

Scheme S2. Synthesis of DeUG coupled silane monomers with fluorinated carbon chain linker.

1. Synthetic procedure

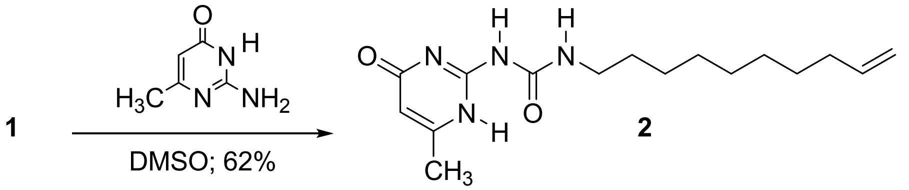

1-(dec-9-enyl)-3-(6-methyl-4-oxo-1,4-dihydropyrimidin-2-yl)urea (2). To a stirred solution of 2-amino-4-hydroxy-6-methylpyrimidine 0.913g (7.30 mmol) in DMSO at 170 °C was added 1 1.53g (8.40 mmol) (1 was synthesized according to a published procedure [1]) under N2. After 3 min, the reaction mixture was quenched in an ice-water bath. The resulting solid was filtered and washed with 15 mL cyclohexane then 20 mL acetone. Crude product was purified by silica gel column chromatography step gradient from (CHCl3:AcOEt, 5:1 v/v) to ($CHCl_3$:AcOEt, 3:1 v/v) afforded 2 (1.39g, 62%) as white solid. mp 151-152 °C; TLC ($CHCl_3$:AcOEt, 3:1 v/v): Rf =0.34; ^{1}H NMR (500 MHz, $CDCl_3$): δ 13.13 (s, 1H), 11.85 (s, 1H), 10.15 (t, J = 5.0, 1H), 5.81 (s, 1H), 5.77-5.83 (m, 1H), 4.90-4.99 (m, 2H), 3.23 (q, J = 7.0, 2H), 2.22 (s, 3H), 2.00-2.04 (m, 2H), 1.57-1.59 (m, 2H), 1.27-1.37 (m, 10H); 13C NMR (126 MHz, $CDCl_3$): δ 173.1, 156.6, 154.8, 148.2, 139.3, 114.2, 106.7, 40.2, 33.9, 29.6, 29.5, 29.3, 29.1, 29.0, 27.0, 19.0; ESI-HR-MS calcd. for $(C_{16}H_{26}N_4O_2+H)^+$, 307.2134; found, 307.2128.

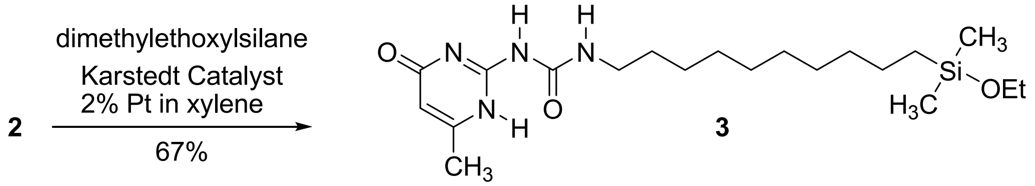

1-(10-(ethoxydimethylsilyl)decyl)-3-(6-methyl-4-oxo-1,4-dihydropyrimidin-2-yl)urea (3). To a 10 mL pear shaped flask with **2** 0.100 g, (0.330 mmol) was added dimethylethoxylsilane 2.0 mL (13.6 mmol) and 15.0 μL Karstedt catalyst (Platinum(0)-1,3-divinyl-1,1,3,3-tetramethyldisiloxane complex solution in xylene, Pt ~2 %). The reaction mixture was refluxed with stirring for 22 h. The open end of condensing column was equipped with a drying tube to prevent any moisture get into the flask. After the reaction, the excessive amount of dimethylethoxylsilane was removed under high vacuum (~0.3 mm Hg) at 50°C and the crude product was further dried in *vacuo* and purified by silica gel column chromatography (MeOH:CH_2Cl_2, 5:95 v/v) to afford **3** (90 mg, 67%) as an white solid. TLC (MeOH:CH_2Cl_2, 5:95 v/v): R_f =0.30; ^{1}H NMR (500 MHz, $CDCl_3$): *δ* 13.12 (s, 1H), 11.82 (s, 1H), 10.13 (bs, 1H), 5.78 (s, 1H), 3.63 (q, *J* = 7.0, 2H), 3.18-3.23 (m, 2H), 2.19 (s, 3H), 1.54-1.58 (m, 2H), 1.23-1.28 (m, 14H), 1.15 (t, *J* = 7.0, 3H), 0.55 (t, *J* = 7.0, 2H), 0.05 (s, 6H); ^{13}C NMR (126 MHz, $CDCl_3$): *δ* 173.4, 156.9, 155.1, 148.5, 107.0, 58.5, 40.4, 33.8, 30.0, 29.9, 29.7, 27.4, 23.5, 19.3, 18.9, 16.7, 16.2, -1.7, -2.3 ; ESI-HR-MS calcd. for $(C_{20}H_{38}N_4O_3Si+H)^+$, 411.2791; found, 411.2786.

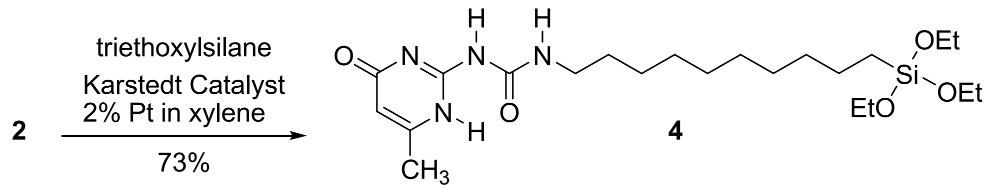

1-(6-methyl-4-oxo-1,4-dihydropyrimidin-2-yl)-3-(10-(triethoxysilyl)decyl)urea (4). To a 10 mL pear shaped flask with **2** 0.124 g (0.404 mmol) was added triethoxylsilane 2.6 mL (13.4 mmol) and 24.0.0 µL Karstedt catalyst (Platinum(0)-1,3-divinyl-1,1,3,3-tetramethyldisiloxane complex solution in xylene, Pt ~2 %). The reaction mixture was heated with stirring at 80°C for 22 h. The open end of condensing column was equipped with a drying tube to prevent any moisture get into the flask. After the reaction, the excessive amount of triethoxylsilane was removed under high vacuum (~0.3 mm Hg) at 50°C and the crude product was further dried in *vacuo* and purified by silica gel column chromatography (MeOH: CH_2Cl_2, 5:95 v/v) to afford **4** (0.138 g, 73%) as an white solid. TLC (MeOH:CH_2Cl_2, 5:95 v/v): R_f =0.25; ^{1}H NMR (500 MHz, $CDCl_3$): δ 13.13 (s, 1H), 11.84 (s, 1H), 10.14 (bs, 1H), 5.80 (s, 1H), 3.80 (q, J = 7.0, 6H), 3.21 (q, J = 6.5, 2H), 2.21 (s, 3H), 1.54-1.59 (m, 2H), 1.33-1.39 (m, 2H),1.19-1.29 (m, 12H), 1.21 (t, J = 7.0, 9H), 0.59-0.63 (m, 2H); ^{13}C NMR (126 MHz, $CDCl_3$): δ 173.2, 156.7, 154.8, 148.3, 106.8, 58.5, 40.2, 33.3, 29.7, 29.6, 29.4, 27.1, 22.9, 19.3, 19.0, 18.4, 10.5; ESI-HR-MS calcd. for $(C_{22}H_{42}N_4O_5Si+H)^+$, 471.3003; found, 471.2999.

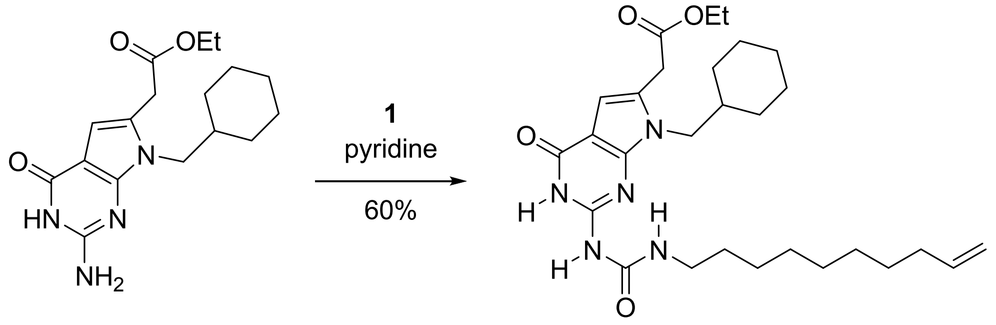

Ethyl 2-(7-(cyclohexylmethyl)-2-(3-dec-9-enylureido)-4-oxo-4,7-dihydro-3H-pyrrolo[2,3-d]pyrimidin-6-yl)acetate (6). To a stirred solution of **5** 1.00g (3.00 mmol) (**5** was synthesized according to a published procedure [2]) in 15 mL pyridine at 95 °C was added **1** 3.26 g (18.0 mmol) via an additional funnel under N_2. The reaction was stirring at 95 °C for 16h and cooled to rt. Crude product was concentrated in *vacuo* and purified by silica gel column chromatography (MeOH:CH_2Cl_2, 5:95 v/v) to afford **6** (0.935 g, 60%) as buff-colored solid. mp: 118-119 °C; TLC (MeOH:CH_2Cl_2, 5:95 v/v): R_f =0.19; ^{1}H NMR (500 MHz, $CDCl_3$): δ 11.27 (s, 1H), 9.44 (s, 1H), 8.99 (bs, 1H), 6.85 (s, 1H), 5.74-5.82 (m, 1H), 4.89-4.99 (m, 2H), 4.16 (q, J = 7.0, 2H), 3.83 (d, J = 8.0, 2H), 3.71 (s, 2H), 4.43 (q, J = 6.5, 2H), 1.99-2.03 (m, 2H), 1.61-1.72 (m, 6H), 1.56-1.59 (m, 2H), 1.29-1.43 (m, 7H) , 1.26 (t, J = 7.0, 3H), 1.12-1.17 (m, 2H), 0.92-0.98 (m, 2H); ^{13}C NMR (126 MHz, $CDCl_3$): δ 170.0, 159.3, 154.1, 148.6, 146.4, 139.2, 127.6, 114.2, 103.4, 102.4, 61.5, 49.3, 40.5, 39.0, 33.9,

33.3, 31.2, 30.2, 29.5, 29.4, 29.2, 29.0, 27.2, 26.3, 25.8, 14.2; ESI-HR-MS calcd. for $(C_{28}H_{43}N_5O_4+H)^+$, 514.3393; found, 514.3396.

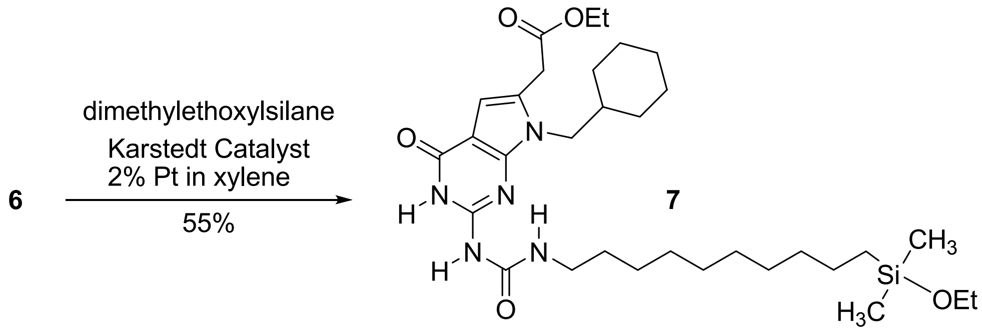

Ethyl 2-(7-(cyclohexylmethyl)-2-(3-(10-(ethoxydimethylsilyl)decyl)ureido)-4-oxo-4,7-dihydro-3H-pyrrolo[2,3-d]pyrimidin-6-yl)acetate (7). To a 10 mL pear shaped flask with **6** 100 mg (0. 195 mmol) was added dimethylethoxylsilane 3.20 mL (21.8 mmol) and 24.0 µL Karstedt catalyst (Platinum(0)-1,3-divinyl-1,1,3,3-tetramethyldisiloxane complex solution in xylene, Pt ~2 %). The reaction mixture was refluxed with stirring for 14 h. The open end of condensing column was equipped with a drying tube to prevent any moisture get into the flask. After the reaction, the excessive amount of dimethylethoxylsilane was removed under high vacuum (~0.3 mm Hg) at 50°C. The crude product was further dried in *vacuo* and purified by silica gel column chromatography (MeOH:CH_2Cl_2, 5:95 v/v) to afford **7** (66 mg, 55%) as a slightly yellow solid. TLC (MeOH:CH_2Cl_2, 5:95 v/v): R_f =0.32; ^{1}H NMR (500 MHz, $CDCl_3$): δ 11.26 (s, 1H), 9.44 (s, 1H), 9.00 (bs, 1H), 6.86 (s, 1H), 4.18 (q, J = 7.0, 2H), 3.84 (d, J = 7.5, 2H), 3.72 (s, 2H), 3.64 (q, J = 7.0, 2H), 3.41-3.46 (m, 2H), 1.62-1.73 (m, 6H), 1.57-1.60 (m, 2H), 1.39-1.44 (m, 2H), 1.30-1.32 (m, 11H), 1.27 (t, J = 7.0, 3H), 1.17 (t, J = 7.0, 3H), 1.12-1.16 (m, 2H), 0.93-0.97 (m, 2H), 0.08 (s, 6H); ^{13}C NMR (126 MHz, $CDCl_3$): δ 170.0, 159.4, 154.1, 148.7, 146.4, 127.6, 103.5, 102.5, 61.5, 58.3, 49.4, 40.6, 39.0, 33.6, 33.4, 31.3, 30.3, 29.8, 29.7, 29.6, 29.5, 27.4, 26.4, 25.9, 23.3, 18.7, 16.5, 14.3, -1.9; ESI-HR-MS calcd. for $(C_{32}H_{55}N_5O_5Si+H)^+$, 618.4051; found, 618.4050.

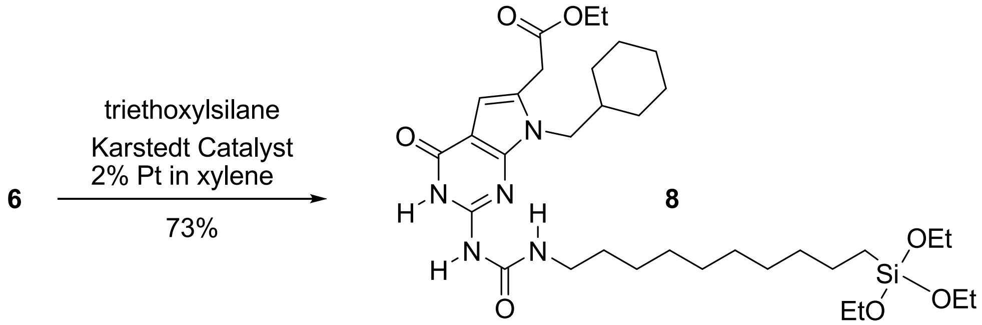

Ethyl 2-(7-(cyclohexylmethyl)-4-oxo-2-(3-(10-(triethoxysilyl)decyl)ureido)-4,7-dihydro-3H-pyrrolo[2,3-d]pyrimidin-6-yl)acetate (8). To a 10 mL pear shaped flask with **6** 124 mg (0.404 mmol) was added

triethoxylsilane 2.6 mL (13.4 mmol) and 24.0.0 μL Karstedt catalyst (Platinum(0)-1,3-divinyl-1,1,3,3-tetramethyldisiloxane complex solution in xylene, Pt ~2 %). The reaction mixture was heated with stirring at 80°C for 22 h. The open end of condensing column was equipped with a drying tube to prevent any moisture get into the flask. After the reaction, the excessive amount of triethoxylsilane was removed under high vacuum (~0.3 mm Hg) at 50°C and the crude product was further dried in *vacuo* and purified by silica gel column chromatography (MeOH:CH_2Cl_2, 5:95 v/v) to afford **8** (0.138 g, 73%) as an white solid. TLC (MeOH:CH_2Cl_2, 5:95 v/v): R_f =0.25; ^{1}H NMR (500 MHz, $CDCl_3$): δ 11.26 (s, 1H), 9.44 (s, 1H), 9.01 (bs, 1H), 6.86 (s, 1H), 4.18 (q, J = 7.0, 2H), 3.84 (d, J = 7.5, 2H), 3.81 (q, J = 7.0, 6H), 3.72 (s, 2H), 3.42-3.45 (m, 2H), 1.92-1.99 (m, 2H), 1.57-1.72 (m, 8H), 1.37-1.42 (m, 2H), 1.32-1.35 (m, 2H), 1.29 (t, J = 7.0, 3H), 1.22 (t, J = 7.0, 9H), 1.12-1.19 (m, 8H), 0.93-0.99 (m, 2H), 0.84-0.92 (m, 1H), 0.62 (t, J = 7.5, 2H); ^{13}C NMR (126 MHz, $CDCl_3$): δ 170.0, 159.3, 154.1, 148.7, 146.4, 127.6, 103.5, 102.5, 61.5, 58.4, 49.4, 40.6, 39.0, 33.4, 32.7, 31.3, 30.3, 29.7, 29.6, 29.4, 27.4, 27.3, 26.4, 25.9, 22.9, 18.4, 14.3, 10.5; ESI-HR-MS calcd. for $(C_{34}H_{59}N_5O_7Si+H)^+$, 678.4262; found, 678.4263.

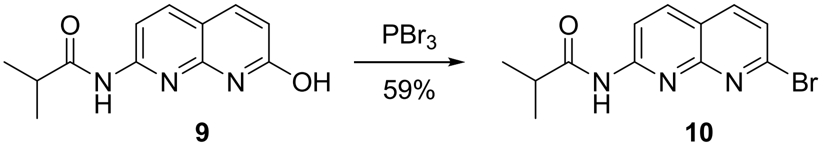

9 **10**

***N*-(7-bromo-1,8-naphthyridin-2-yl)isobutyramide (10).** To a Schlenk flask with **9** 2.72g (11.8 mmol) (**9** was synthesized according to a published procedure [3]) was added PBr_3 (22.7 mL, 242 mmol) via syringe under N_2. The reaction mixture was heated to 110 °C for 24 h. The crude mixture was cooled in ice bath and extracted with ($CHCl_3$:isopropanol, 9:1 v/v) (4 × 100 mL). The combined organic layer was washed with saturated aqueous sodium bicarbonate (2 × 50 mL) and water (2 × 100 mL). The organic layer was dried over sodium sulfate, concentrated in *vacuo* and purified by silica gel column chromatography step gradient from (CH_2Cl_2:conc. aq. NH_4OH, 99:1 v/v) to (CH_2Cl_2:MeOH:conc. aq NH_4OH, 90:10:1 v/v/v) to afford **10** (2.06 g, 59%) as a white powder. mp 185–186 °C; TLC (CH_2Cl_2:MeOH:conc. aq NH_4OH, 90:10:1 v/v/v): R_f =0.67; ^{1}H NMR (500 MHz; DMSO-d_6): δ 9.52 (s, 1H), 8.56 (d, J = 9.0, 2H), 8.16 (d, J = 9.0, 1H), 7.93 (d, J = 8.0, 1H), 7.48 (d, J = 8.5, 1H), 2.77 (septet, J = 7.0, 1H), 1.21 (d, J = 7.0, 6H); ^{13}C NMR (126 MHz; DMSO-d_6): δ 177.0, 154.9, 154.6, 145.3, 139.4, 138.3, 125.5, 119.3, 116.1, 36.5, 19.4; ESI-HR-MS calcd. for $(C_{12}H_{12}BrN_3O+H)^+$, 294.0242; found, 294.0234.

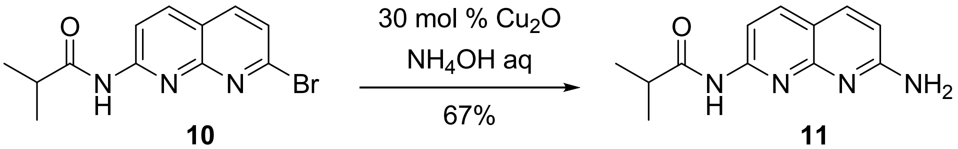

10 **11**

***N*-(7-amino-1,8-naphthyridin-2-yl)isobutyramide (11).** To a 15 mL thick-walled, high-pressure tube with magnetic stir-bar and **10** 1.00g (3.40 mmol) was added Cu_2O 0.150g (1.05 mmol) and anhydrous ethylene glycol 4.0 mL. Con. aq NH_4OH 2.40 mL was quickly added followed by immediately seal the tube with a Teflon screw cap. The reaction mixture was stirred at ambient temperature for 24 h and during this time the reaction tube was flipped up-side down and shaking vigorously with hand every 1 h. Interesting color change was observed during the reaction: upon adding NH_4OH, red color, after 30 min, blue color, after 2 h, yellow-brown, after 16h, yellow-green. After 24 h, the reaction tube was opened with caution and crude mixture was diluted with 20 mL CH_2Cl_2/H_2O 1:1 (v/v). The aqueous phase was extracted with (chloroform:isopropanol, 9:1 v/v) (5 × 25 mL). The combined organic layer was washed with water (3 × 25 mL), brine (25 mL), dried over sodium sulfate, concentrated in *vacuo* and purified by silica gel column chromatography (MeOH:CH_2Cl_2, 1:9 v/v) to afford **11** (0.532 g, 67%) as an off-white powder. mp 211–212 °C; TLC (MeOH:CH_2Cl_2, 1:9 v/v): R_f =0.31; ^{1}H NMR (500 MHz; DMSO-d_6): δ 10.56 (s, 1H), 7.97 (s, 2H), 7.81 (d, J = 10.5, 1H), 6.69 (d, J = 8.5, 1H), 6.67 (d, J = 8.0, 2H), 2.78 (septet, J = 7.0, 1H), 1.07 (d, J = 7.0, 6H); ^{13}C NMR (126 MHz; DMSO-d_6): δ 177.2, 161.7, 156.6, 154.1, 138.8, 137.7, 114.6, 111.9, 109.5, 35.3, 20.1; ESI-HR-MS calcd. for $(C_{12}H_{14}N_4O+H)^+$, 231.1246; found, 231.1246.

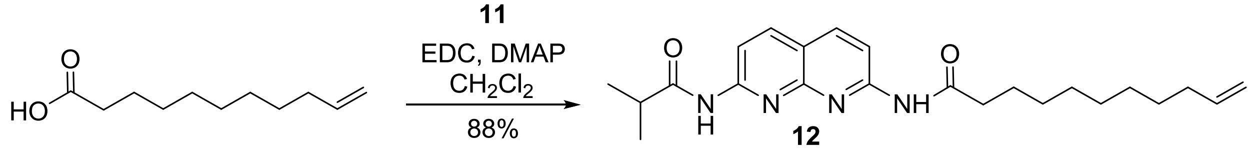

***N*-(7-isobutyramido-1,8-naphthyridin-2-yl)undec-10-enamide (12).** To a stirred solution of **11** (0.300g, 1.29 mmol) in 12.0 mL CH_2Cl_2 was added 4-(dimethylamino)pyridine (DMAP) (47.4 mg, 0.388 mmol), 10-undecenoic acid (0.783 mL 3.87 mmol), the reaction was cooled to 0 °C in an ice-water bath, *N*-(3-dimethylaminopropyl) -*N'*-ethyl carbodiimide hydrochloride (EDC) (0.744g, 3.87 mmol) was added. The ice-water bath was removed and reaction was allowed to warm to ambient temperature and was kept stirring under N_2 for 24 h. The solution was diluted with 100 mL CH_2Cl_2, washed with water (2 × 50 mL), saturated aqueous sodium bicarbonate (2 × 40 mL) and with brine (50 mL). The organic layer was dried over sodium sulfate, concentrated in *vacuo* and purified by silica gel column chromatography (AcOEt:Petroleum ether, 1:1 v/v) to afford **12** (0.606 g, 88%) as white solid. mp 168-169 °C; TLC (AcOEt:Petroleum ether, 1:1 v/v): R_f =0.49; ^{1}H NMR (500 MHz, $CDCl_3$): δ 9.41 (s, 1H), 9.02 (s, 1H), 8.47 (d, J = 9.0, 1H), 8.46 (d, J = 9.0, 1H), 8.05 (d, J = 9.0, 1H), 8.04 (d, J = 9.0, 1H), 5.72-5.80 (m, 1H), 4.88-4.97 (m, 2H), 2.66 (septet, J = 7.0, 1H), 2.42 (t, J = 7.5, 2H), 1.99 (q, J = 7.5, 2H), 1.65 (pentet, J = 7.0, 2H), 1.22-1.23 (d, J = 6.5, 6H), 1.20-1.32 (m, 10H),; ^{13}C NMR (126 MHz, $CDCl_3$): δ 176.5, 172.9, 154.4, 154.3, 153.6, 139.2, 139.1, 118.3, 114.2, 113.8, 113.7, 37.9, 36.7, 33.8, 29.3, 29.2, 29.1, 28.9, 25.3, 19.4; ESI-HR-MS calcd. for $(C_{23}H_{34}N_4O_2+H)^+$, 397.2604; found, 397.2600.

12

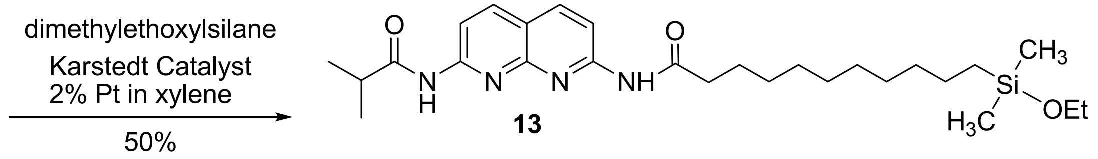

11-(ethoxydimethylsilyl)-*N*-(7-isobutyramido-1,8-naphthyridin-2-yl)undecanamide (13). To a 10 mL pear shaped flask with **12** 100 mg (0. 252 mmol) was added dimethylethoxylsilane 3.60 mL (24.6 mmol) and 28.0 µL Karstedt catalyst (Platinum(0)-1,3-divinyl-1,1,3,3-tetramethyldisiloxane complex solution in xylene, Pt ~2 %). The reaction mixture was refluxed with stirring for 24 h. The open end of condensing column was equipped with a drying tube to prevent any moisture get into the flask. After the reaction, the excessive amount of dimethylethoxylsilane was removed under high vacuum (~0.3 mm Hg) at 50°C and the crude product was further dried in *vacuo* and purified by silica gel column chromatography (AcOEt:Petroleum ether, 1:1 v/v) to afford **13** (63 mg, 50%) as a white solid. TLC (AcOEt:Petroleum ether, 1:1 v/v): R_f =0.79; ^{1}H NMR (500 MHz, $CDCl_3$): δ 8.44 (d, J = 9.0, 1H), 8.42 (d, J = 9.0, 1H), 8.21 (s, 2H), 8.13 (d, J = 9.0, 2H), 3.65 (q, J = 7.0, 2H), 2.62 (septet, J = 7.0, 1H), 2.45 (t, J = 7.0, 2H), 1.72-1.76 (m, 2H), 1.35-1.39 (m, 2H), 1.30 (d, J = 6.5, 6H), 1.26-1.34 (m, 12H), 1.18 (t, J = 7.0, 3H), 0.58 (t, J = 7.5, 2H), 0.08 (s, 6H); ^{13}C NMR (126 MHz, $CDCl_3$): δ 176.1, 172.4, 154.1, 153.9, 153.8, 139.1, 118.5, 113.5, 58.3, 38.2, 37.1, 33.6, 29.6, 29.4, 29.3, 25.4, 23.3, 19.5, 18.7, 16.5, -1.9; ESI-HR-MS calcd. for $(C_{27}H_{44}N_4O_3Si{+}H)^+$, 501.3261; found, 501.3249.

12

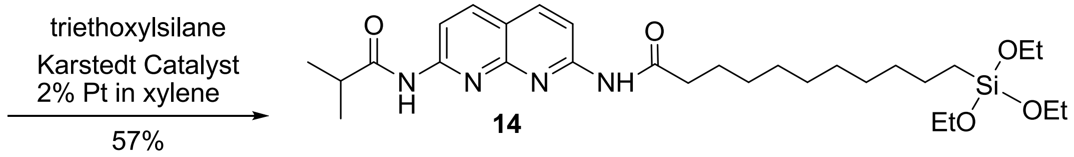

***N*-(7-isobutyramido-1,8-naphthyridin-2-yl)-11-(triethoxysilyl)undecanamide (14).** To a 10 mL pear shaped flask with **12** 100 mg (0.252 mmol) was added triethoxylsilane 3.4 mL (17.5 mmol) and 30.0.0 µL Karstedt catalyst (Platinum(0)-1,3-divinyl-1,1,3,3-tetramethyldisiloxane complex solution in xylene, Pt ~2 %). The reaction mixture was heated with stirring at 100°C for 5 h. The open end of condensing column was equipped with a drying tube to prevent any moisture get into the flask. After the reaction, the excessive amount of triethoxylsilane was removed under high vacuum (~0.3 mm Hg) at 50°C and the crude product was further dried in *vacuo* and purified by silica gel column chromatography ($MeOH{:}CH_2Cl_2$, 5:95 v/v) to afford **14** (81 mg, 57%) as slightly yellow solid. TLC ($MeOH{:}CH_2Cl_2$, 5:95 v/v): R_f =0.42; ^{1}H NMR (500 MHz, $CDCl_3$): δ 9.07 (s, 1H), 8.79 (s, 1H), 8.45 (d, J = 9.0, 2H), 8.08 (d, J = 9.0, 2H), 3.79 (q, J = 7.0, 6H), 2.64 (septet, J = 7.0, 1H), 2.42 (t, J = 7.5, 2H), 1.96-1.99 (m, 2H), 1.64-1.69 (m, 2H), 1.24 (d, J = 6.5, 6H), 1.20-1.39 (m, 12H), 1.19 (t, J = 7.0, 9H), 0.59 (t, J = 7.5, 2H); ^{13}C NMR (126 MHz, $CDCl_3$): δ 176.4, 172.7, 154.2, 153.7, 139.2, 139.1, 118.3, 113.6, 58.4, 38.0, 37.9, 36.9, 33.8, 33.3, 29.6, 29.4, 29.3, 29.2, 29.1, 29.0, 25.3, 22.9, 19.4, 18.4, 10.5; ESI-HR-MS calcd. for $(C_{29}H_{48}N_4O_5Si{+}H)^+$, 561.3472; found, 561.3477.

2. ^{1}H and ^{13}C NMR Spectra

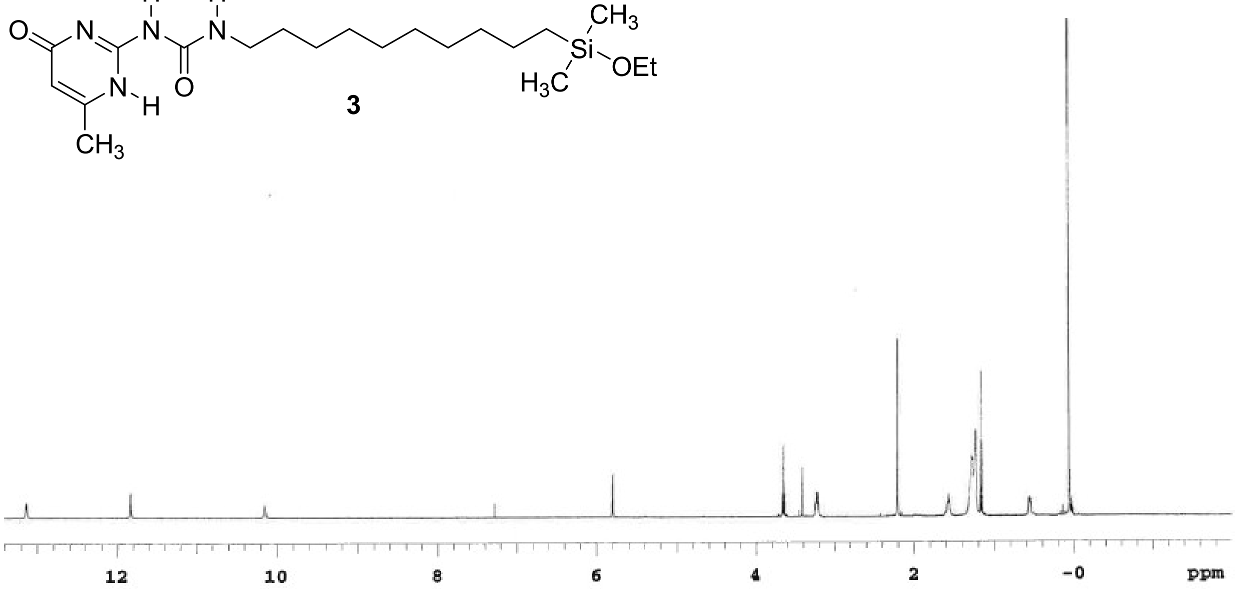

Figure S1. Compound 3, ^{1}H NMR (500 MHz, $CDCl_3$)

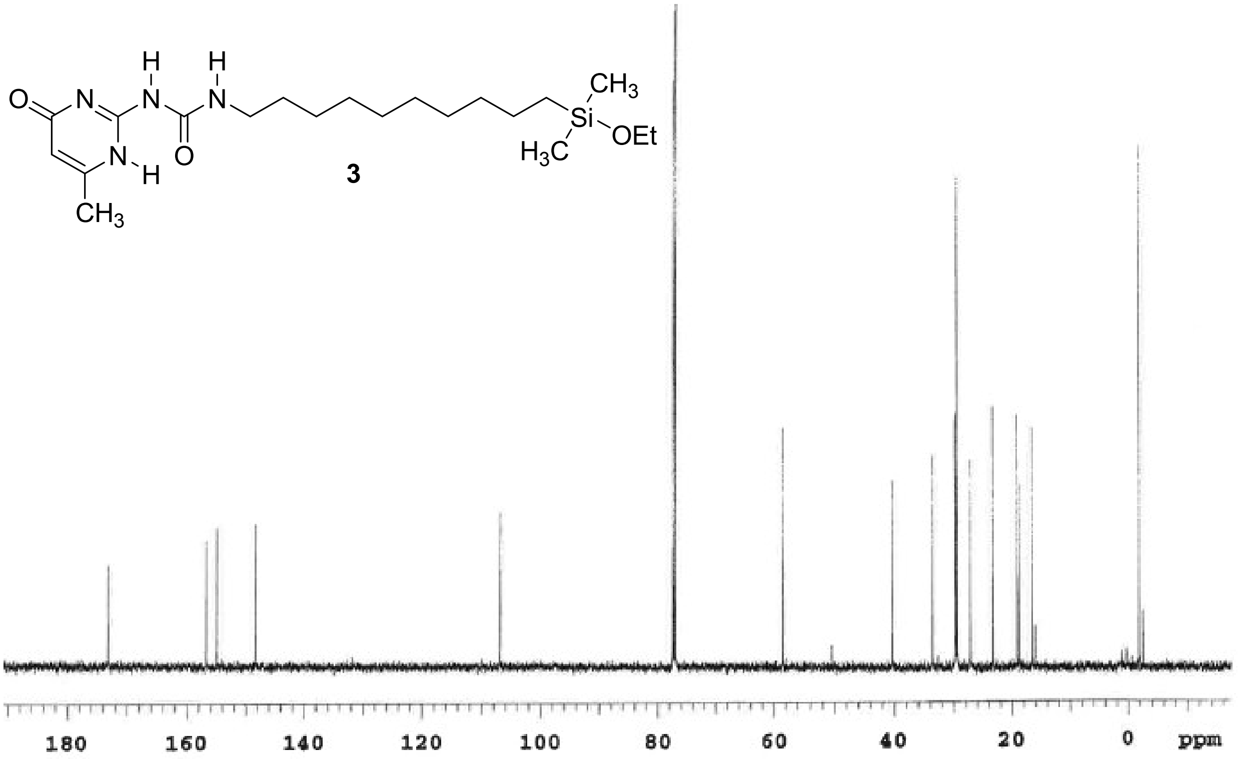

Figure S2. Compound 3, ^{13}C NMR (126 MHz, $CDCl_3$)

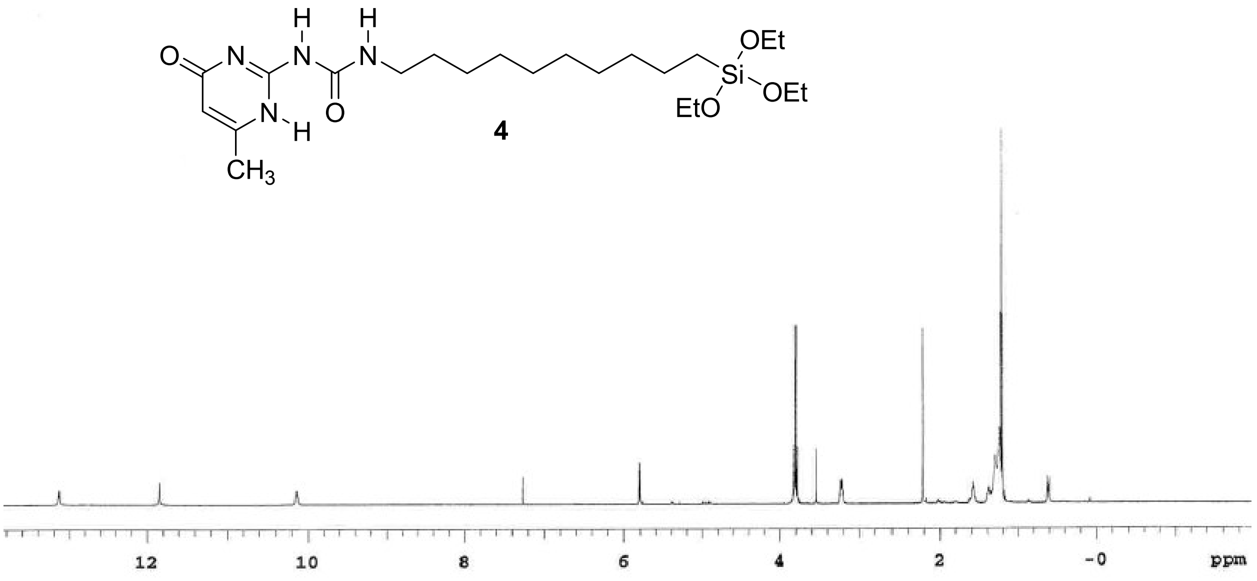

Figure S3. Compound 4, ^{1}H NMR (500 MHz, $CDCl_3$)

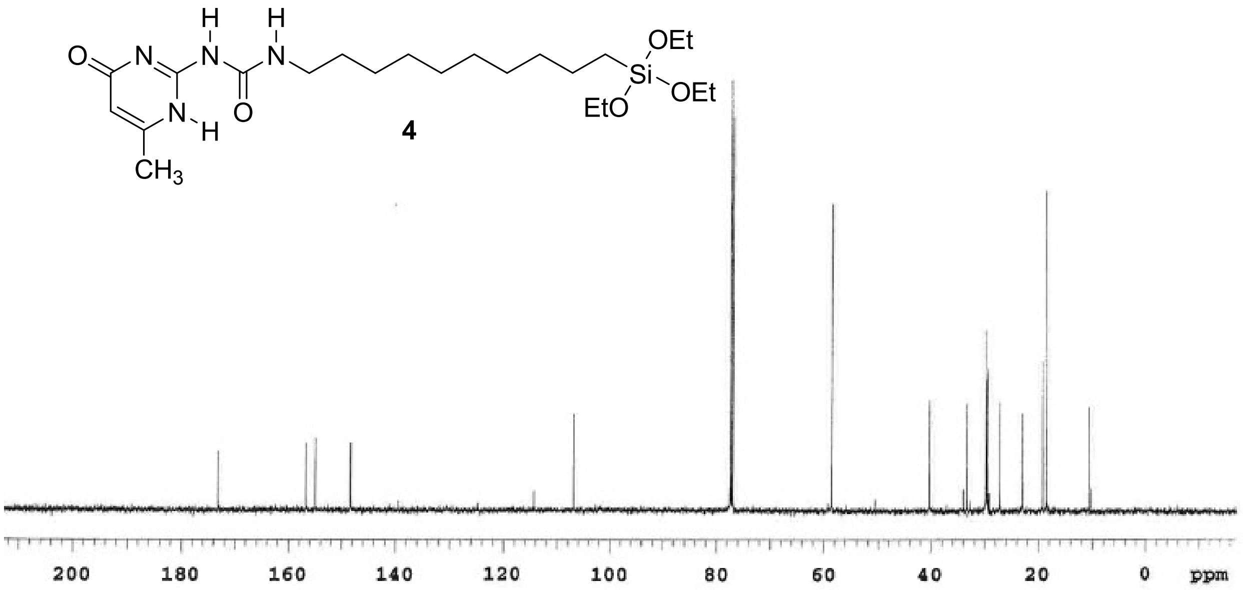

Figure S4. Compound 4, ^{13}C NMR (126 MHz, $CDCl_3$)

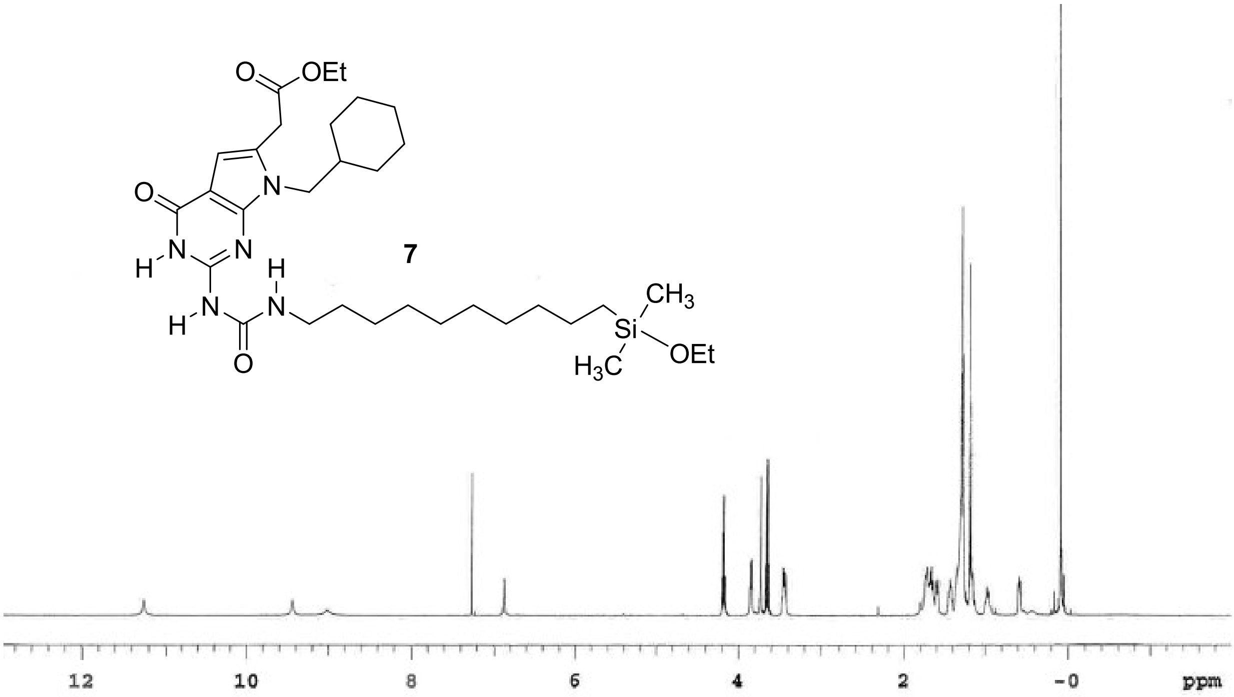

Figure S5. Compound 7, ^{1}H NMR (500 MHz, $CDCl_3$)

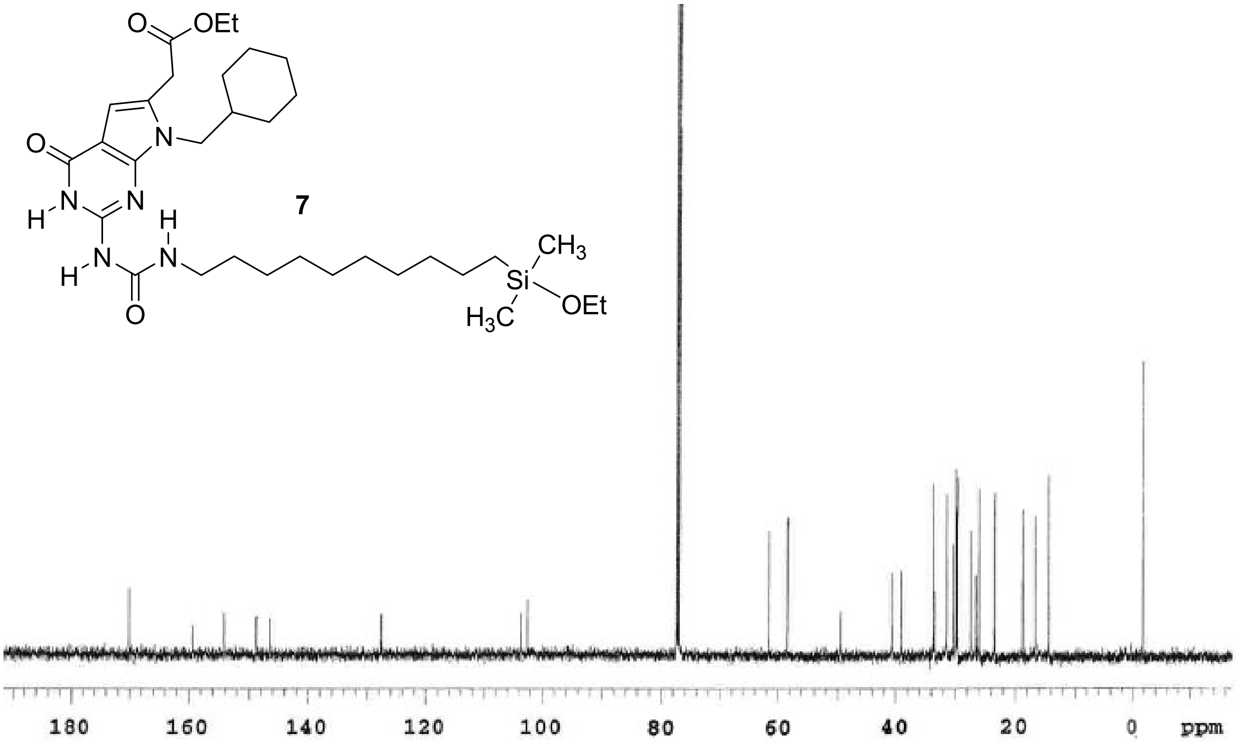

Figure S6. Compound 7, ^{13}C NMR (126 MHz, $CDCl_3$)

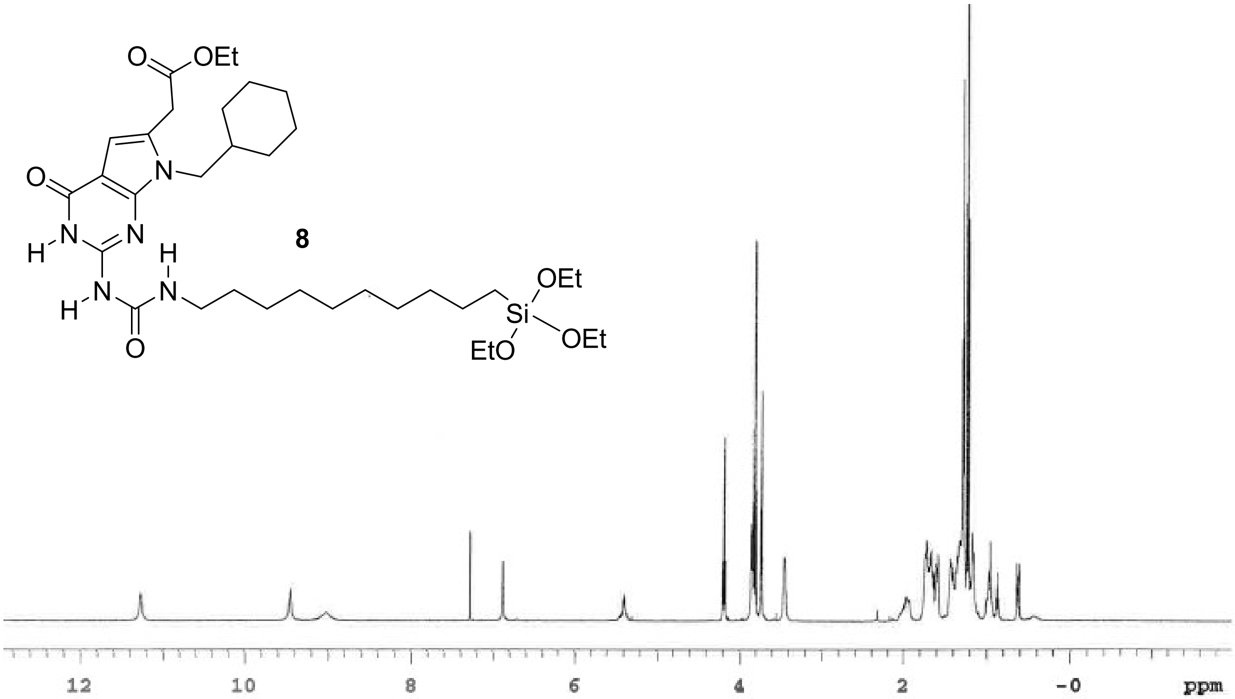

Figure S7. Compound 8, ^{1}H NMR (500 MHz, CDCl$_3$)

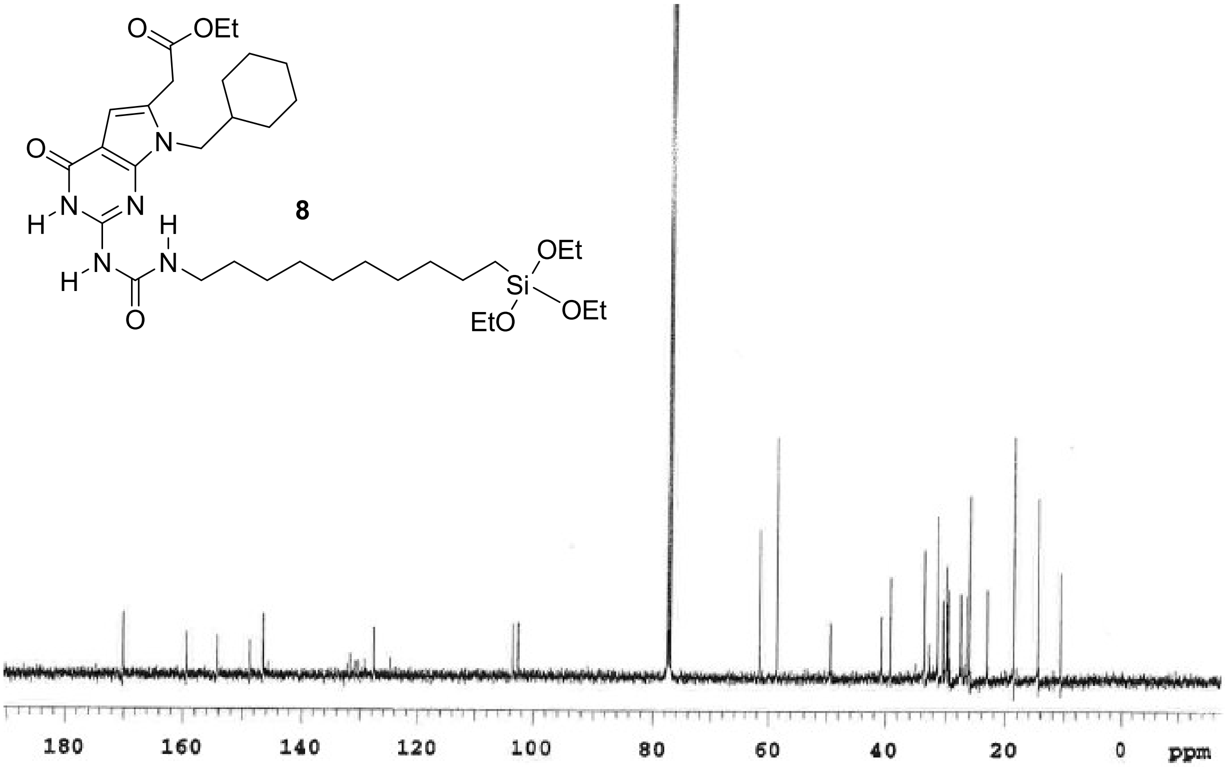

Figure S8. Compound 8, ^{13}C NMR (126 MHz, CDCl$_3$)

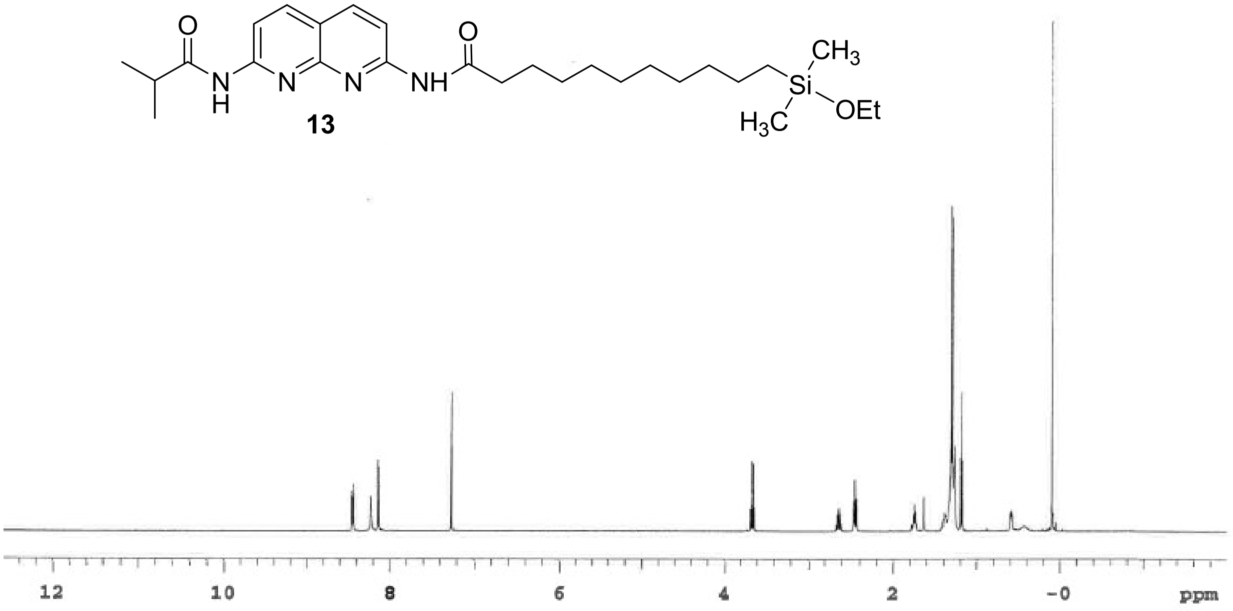

Figure S9. Compound 13, ^{1}H NMR (500 MHz, $CDCl_3$)

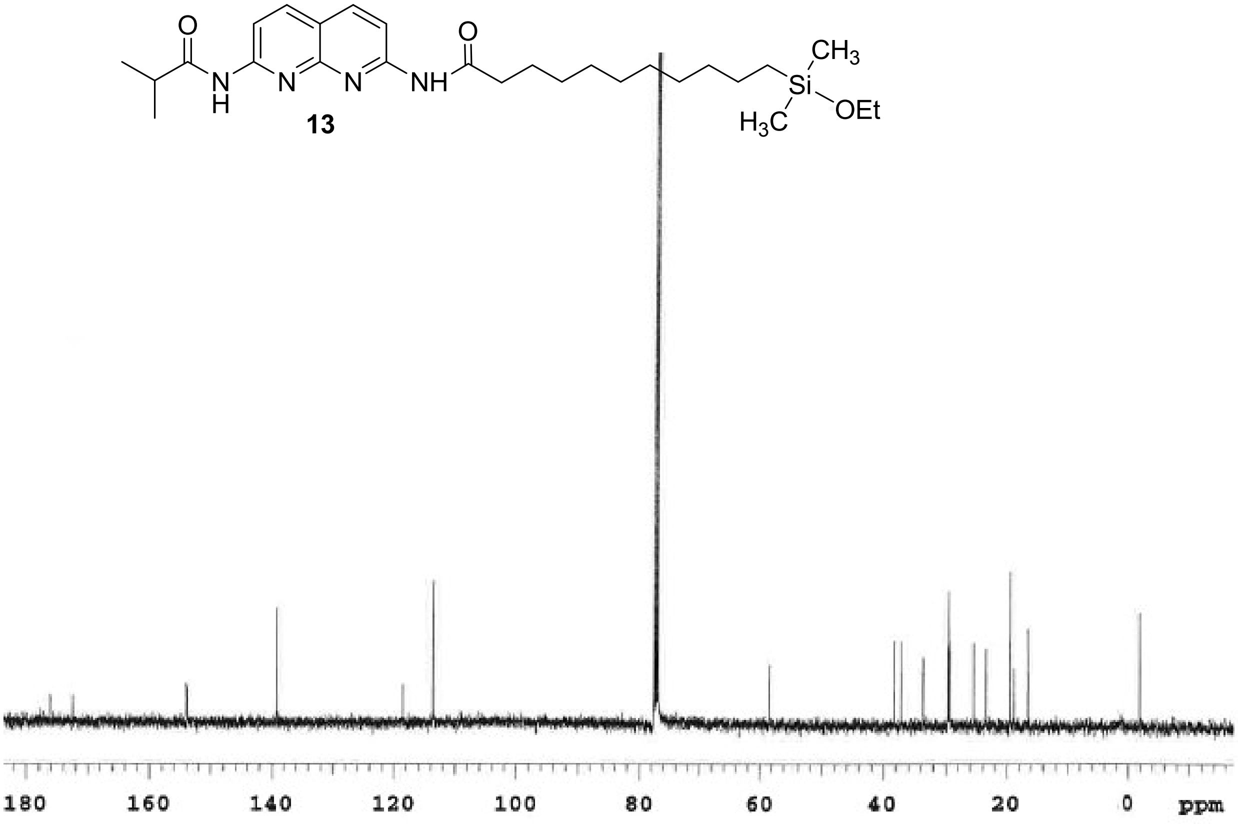

Figure S10. Compound 13, ^{13}C NMR (126 MHz, $CDCl_3$)

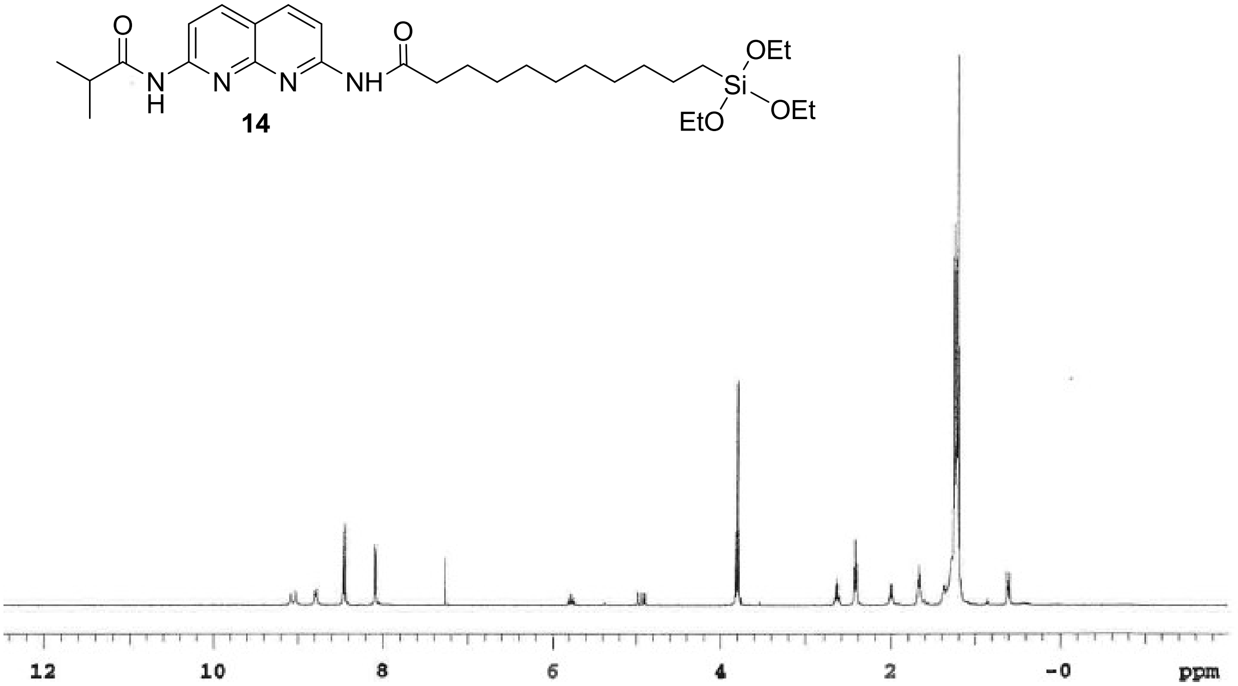

Figure S11. Compound 14, ^1H NMR (500 MHz, $CDCl_3$)

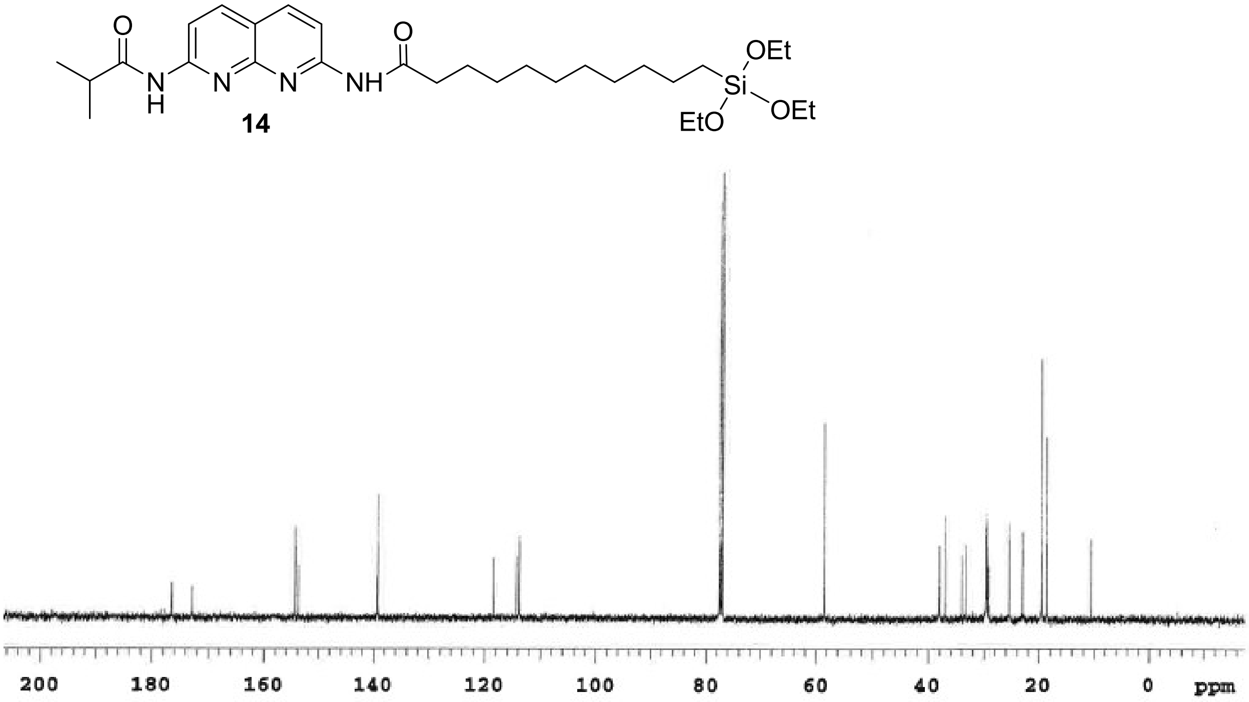

Figure S12. Compound 14, ^{13}C NMR (126 MHz, $CDCl_3$)

3. Elemental composition report of HR-ESI-MS

Elemental Composition Report — **Page 1**

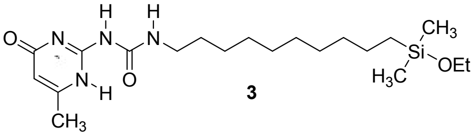

Single Mass Analysis
Tolerance = 10.0 PPM / DBE: min = -1.5, max = 150.0
Element prediction: Off
Number of isotope peaks used for i-FIT = 3

Monoisotopic Mass, Even Electron Ions
398 formula(e) evaluated with 3 results within limits (all results (up to 1000) for each mass)
Elements Used:
C: 0-150 H: 0-250 N: 0-5 O: 0-6 Na: 0-1 Si: 1-1
Zhang, Yagang, scz-zyg-2-181 University of Illinois, SCS, Mass Spectrometry Lab Q-tof UE521
Qtof_34343B 44 (3.150) AM (Cen,3, 80.00, Ar,15000.0,716.46,0.70,LS 3); Sm (SG, 2x3.00); Cm (44:45) 1: TOF MS ES+ 5.09e+003

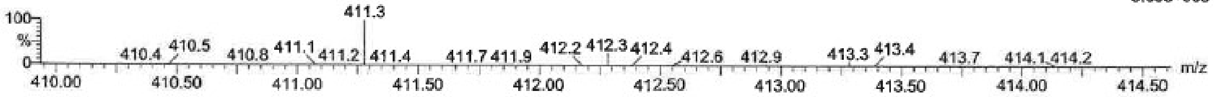

Mass	Calc. Mass	mDa	PPM	DBE	i-FIT	Formula
Minimum:				-1.5		
Maximum:		5.0	10.0	150.0		
411.2786	411.2791	-0.5	-1.2	4.5	6.0	C20 H39 N4 O3 Si
	411.2767	1.9	4.6	1.5	8.4	C18 H40 N4 O3 Na Si
	411.2808	-2.2	-5.3	5.5	15.3	C23 H40 N2 O Na Si

Figure S13. Elemental composition report of compound 3. (HR-ESI-MS)

Elemental Composition Report — **Page 1**

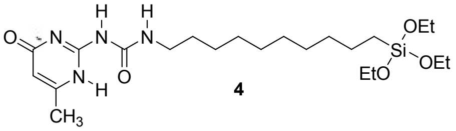

Single Mass Analysis
Tolerance = 10.0 PPM / DBE: min = -1.5, max = 150.0
Element prediction: Off
Number of isotope peaks used for i-FIT = 3

Monoisotopic Mass, Even Electron Ions
238 formula(e) evaluated with 2 results within limits (all results (up to 1000) for each mass)
Elements Used:
C: 0-150 H: 0-250 N: 0-5 O: 0-6 Si: 1-1
Zhang, Yagang, scz-zyg-2-193 University of Illinois, SCS, Mass Spectrometry Lab Q-tof UE521
Qtof_34563 25 (1.793) AM (Cen,3, 80.00, Ar,15000.0,716.46,0.70,LS 3); Sm (SG, 2x5.00); Cm (25:27) 1: TOF MS ES+ 1.68e+003

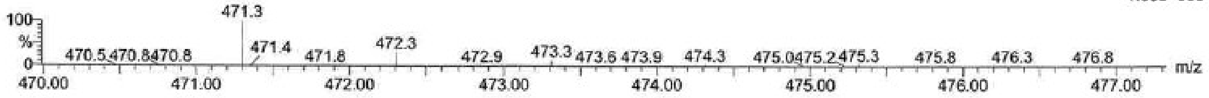

Mass	Calc. Mass	mDa	PPM	DBE	i-FIT	Formula
Minimum:				-1.5		
Maximum:		5.0	10.0	150.0		
471.2999	471.3003	-0.4	-0.8	4.5	5.3	C22 H43 N4 O5 Si
	471.3043	-4.4	-9.3	8.5	11.1	C27 H43 N2 O3 Si

Figure S14. Elemental composition report of compound 4. (HR-ESI-MS)

Elemental Composition Report

Page 1

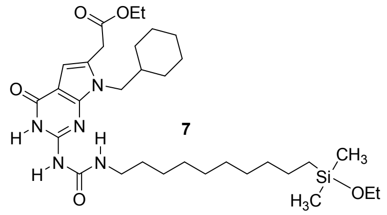

7

Single Mass Analysis

Tolerance = 10.0 PPM / DBE: min = -1.5, max = 150.0
Element prediction: Off
Number of isotope peaks used for i-FIT = 3

Monoisotopic Mass, Even Electron Ions
526 formula(e) evaluated with 6 results within limits (all results (up to 1000) for each mass)
Elements Used:
C: 0-150 H: 0-250 N: 0-5 O: 0-5 Na: 0-1 Si: 1-1

Zhang, Yagang, scz-zyg-2-203-3 University of Illinois, SCS, Mass Spectrometry Lab Q-tof UE521
Qtof_34641A 20 (1.436) AM (Cen,3, 80.00, Ar,15000.0,716.46,0.70,LS 3); Sm (SG, 2x5.00); Cm (20:21) 1: TOF MS ES+ 5.21e+002

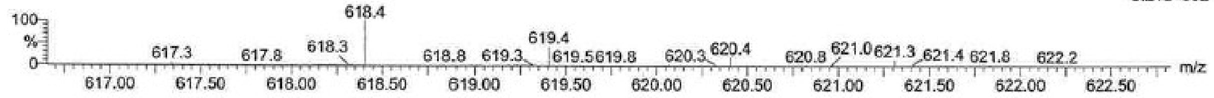

Minimum:				-1.5		
Maximum:		5.0	10.0	150.0		
Mass	Calc. Mass	mDa	PPM	DBE	i-FIT	Formula
618.4050	618.4051	-0.1	-0.2	8.5	4.4	C32 H56 N5 O5 Si
	618.4067	-1.7	-2.7	9.5	4.3	C35 H57 N3 O3 Na Si
	618.4027	2.3	3.7	5.5	5.5	C30 H57 N5 O5 Na Si
	618.4091	-4.1	-6.6	12.5	4.4	C37 H56 N3 O3 Si
	618.4107	-5.7	-9.2	13.5	5.3	C40 H57 N O Na Si
	618.3992	5.8	9.4	17.5	5.5	C39 H52 N5 Si

Figure S15. Elemental composition report of compound 7. (HR-ESI-MS)

Elemental Composition Report

Page 1

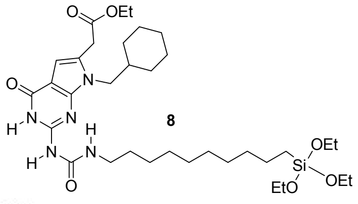

8

Single Mass Analysis

Tolerance = 10.0 PPM / DBE: min = -1.5, max = 150.0
Element prediction: Off
Number of isotope peaks used for i-FIT = 3

Monoisotopic Mass, Even Electron Ions
56 formula(e) evaluated with 2 results within limits (all results (up to 1000) for each mass)
Elements Used:
C: 0-150 H: 0-250 N: 5-6 O: 6-7 Na: 0-1 Si: 1-1

Zhang, Yagang, scz-zyg-2-205 University of Illinois, SCS, Mass Spectrometry Lab Q-tof UE521
Qtof_34670 25 (1.793) AM (Cen,3, 80.00, Ar,15000.0,716.46,0.70,LS 3); Sm (SG, 2x3.00); Cm (25:27) 1: TOF MS ES+ 2.08e+003

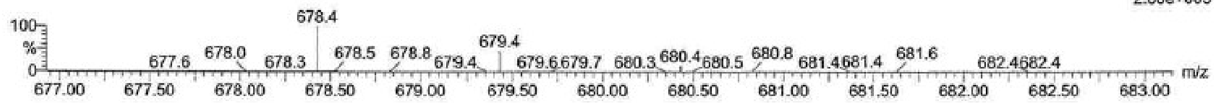

Minimum:				-1.5		
Maximum:		5.0	10.0	150.0		
Mass	Calc. Mass	mDa	PPM	DBE	i-FIT	Formula
678.4263	678.4262	0.1	0.1	8.5	1.4	C34 H60 N5 O7 Si
	678.4238	2.5	3.7	5.5	1.6	C32 H61 N5 O7 Na Si

Figure S16. Elemental composition report of compound 8. (HR-ESI-MS)

Elemental Composition Report **Page 1**

Single Mass Analysis
Tolerance = 10.0 PPM / DBE: min = -1.5, max = 150.0
Element prediction: Off
Number of isotope peaks used for i-FIT = 3

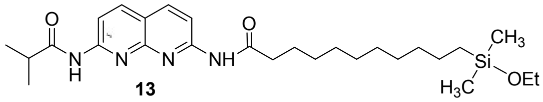

13

Monoisotopic Mass, Even Electron Ions
253 formula(e) evaluated with 1 results within limits (all results (up to 1000) for each mass)
Elements Used:
C: 0-150 H: 0-250 N: 0-5 O: 0-6 Si: 1-1
Zhang, Yagang, scz-zyg-2-191 University of Illnois, SCS, Mass Spectrometry Lab Q-tof UE521
Qtof_34526A 18 (1.293) AM (Cen,3, 80.00, Ar,15000.0,716.46,0.70,LS 3); Sm (SG, 2x5.00) 1: TOF MS ES+ 1.68e+003

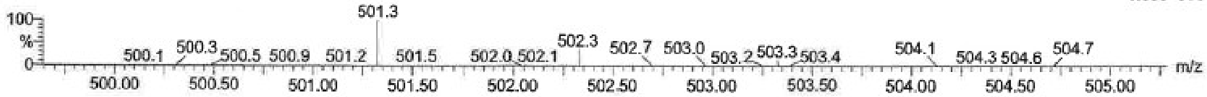

Minimum:				-1.5		
Maximum:		5.0	10.0	150.0		
Mass	Calc. Mass	mDa	PPM	DBE	i-FIT	Formula
501.3249	501.3261	-1.2	-2.4	8.5	3.0	C27 H45 N4 O3 Si

Figure S17. Elemental composition report of compound 13. (HR-ESI-MS)

Elemental Composition Report **Page 1**

Single Mass Analysis
Tolerance = 10.0 PPM / DBE: min = -1.5, max = 150.0
Element prediction: Off
Number of isotope peaks used for i-FIT = 3

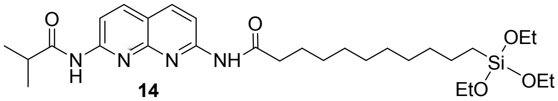

14

Monoisotopic Mass, Even Electron Ions
327 formula(e) evaluated with 2 results within limits (all results (up to 1000) for each mass)
Elements Used:
C: 0-150 H: 0-250 N: 0-6 O: 0-6 Si: 1-1
Zhang, Yagang, scz-zyg-2-249 University of Illnois, SCS, Mass Spectrometry Lab Q-tof UE521
Qtof_35431 31 (2.221) AM (Cen,3, 80.00, Ar,15000.0,716.46,0.70,LS 3); Sm (SG, 2x3.00); Cm (31:34) 1: TOF MS ES+ 2.59e+003

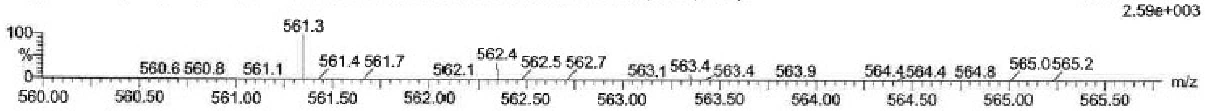

Minimum:				-1.5		
Maximum:		5.0	10.0	150.0		
Mass	Calc. Mass	mDa	PPM	DBE	i-FIT	Formula
561.3477	561.3472	0.5	0.9	8.5	1.7	C29 H49 N4 O5 Si
	561.3512	-3.5	-6.2	12.5	11.3	C34 H49 N2 O3 Si

Figure S18. Elemental composition report of compound 14. (HR-ESI-MS)

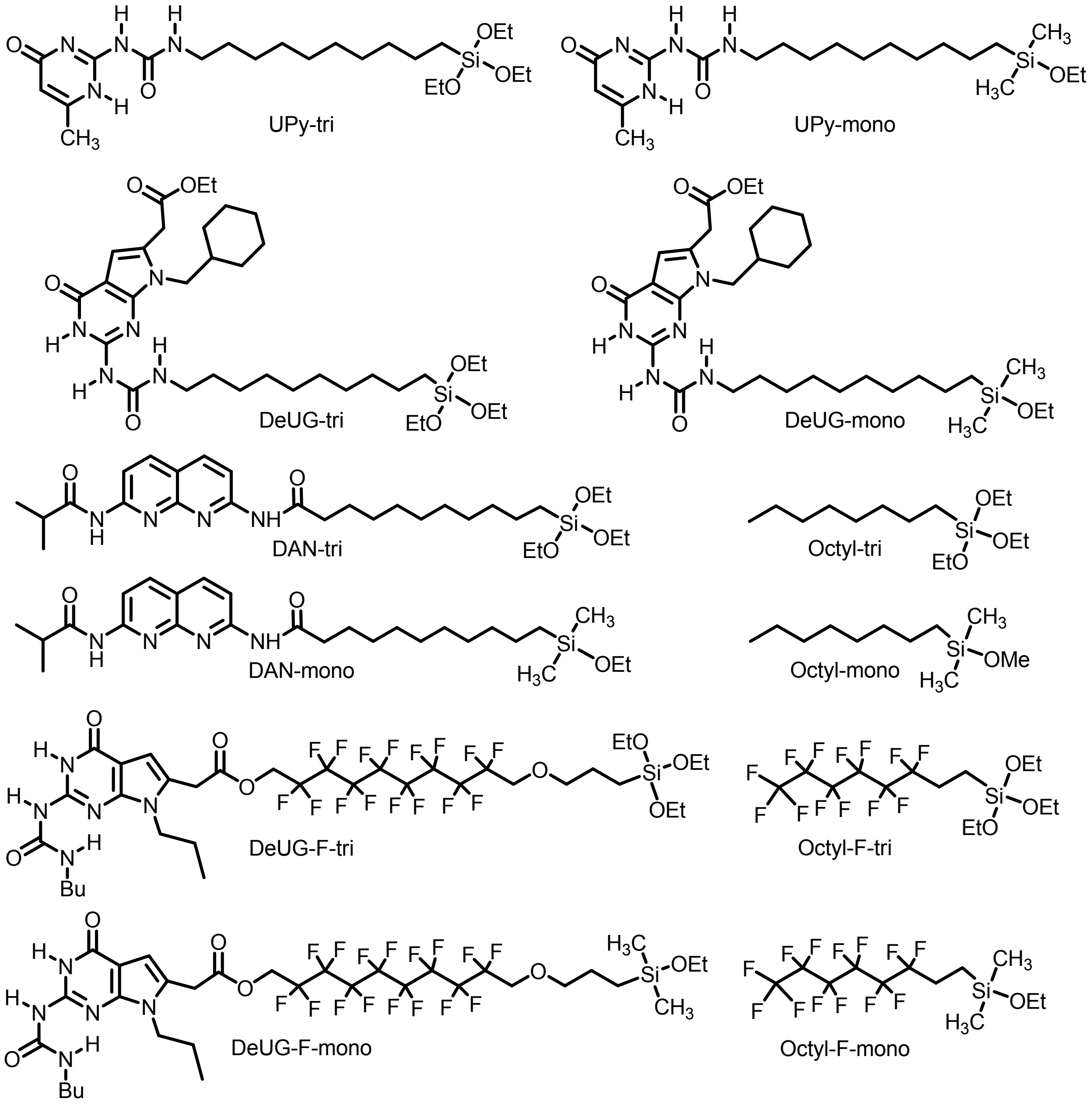

Figure S19. QHB modules coupled silane monomers for surface modification and their abbreviations.

4. Static water contact angle measurement

Table S1. Static water contact angle of unmodified and modified glass slides with QHB coupled silane monomers

**Surface**	**Static water contact angle**
Oxidized glass surface	10±2.0 °
UPy-tri	54.3±1.7 °
DeUG-tri	58.0±1.9 °
DAN-tri	62.5±1.6 °
Octyl-tri	92.8±1.6 °
(UPy+Octyl)-tri	63.9±2.1 °
(DeUG+Octyl)-tri	66.0±1.8 °
(DAN+Octyl)-tri	70.2±2.3 °
UPy-mono	52.2±2.2 °
DeUG-mono	56.6±2.5 °
DAN-mono	59.3±1.8 °
Octyl-mono	83.5±3.1 °
(UPy+Octyl)-mono	65.2±2.4 °
(DeUG+Octyl)-mono	63.6±1.8 °
(DAN+Octyl)-mono	68.8±2.0 °
DeUG-F-tri	71.0±1.5 °
DeUG-F-mono	67.3±1.9 °
Octyl-F-tri	116.5±1.8 °
Octyl-F-mono	91.6±2.6 °
(DeUG-F-tri)+(Octyl-F-tri)	78.2±2.1 °
(DeUG-F-mono)+(Octyl-F-mono)	73.1±2.2 °

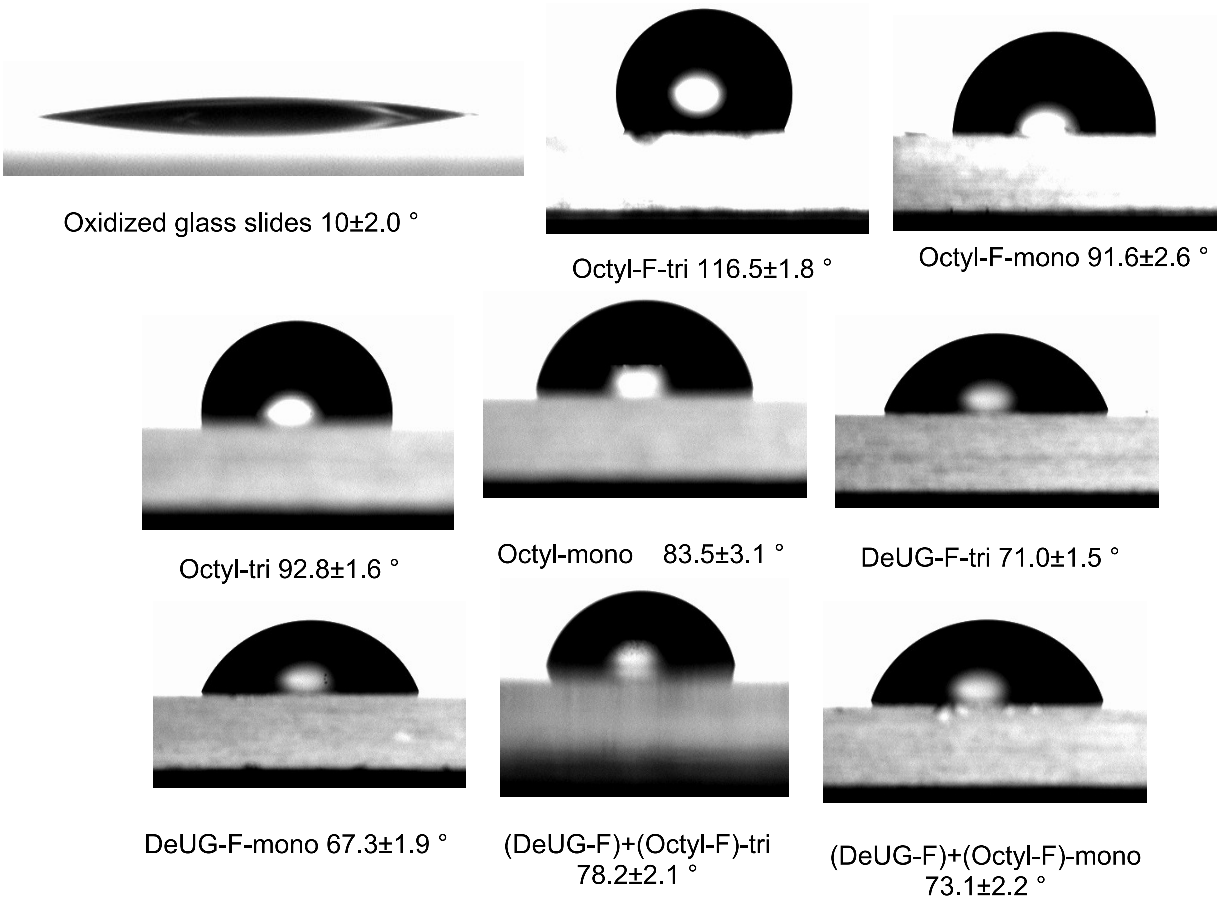

Figure S20. Static water contact angle of unmodified and modified glass slides with QHB coupled silane monomers with fluorinated alkyl linker.

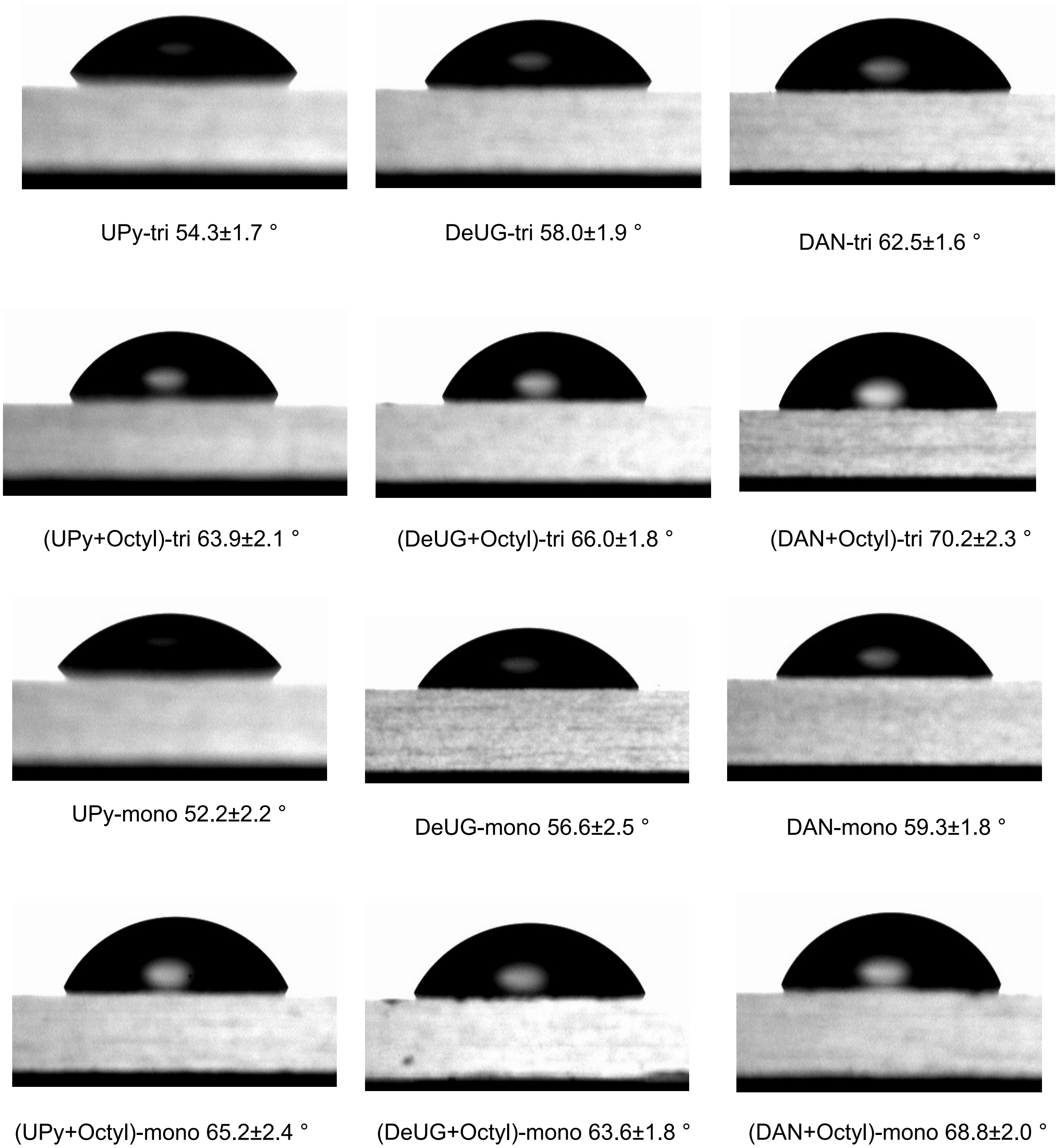

Figure S21. Static water contact angle of modified glass slides with QHB coupled silane monomers, mono-layer and mixed mono-layer.

5. MALDI-TOF-MS of modified glass slides

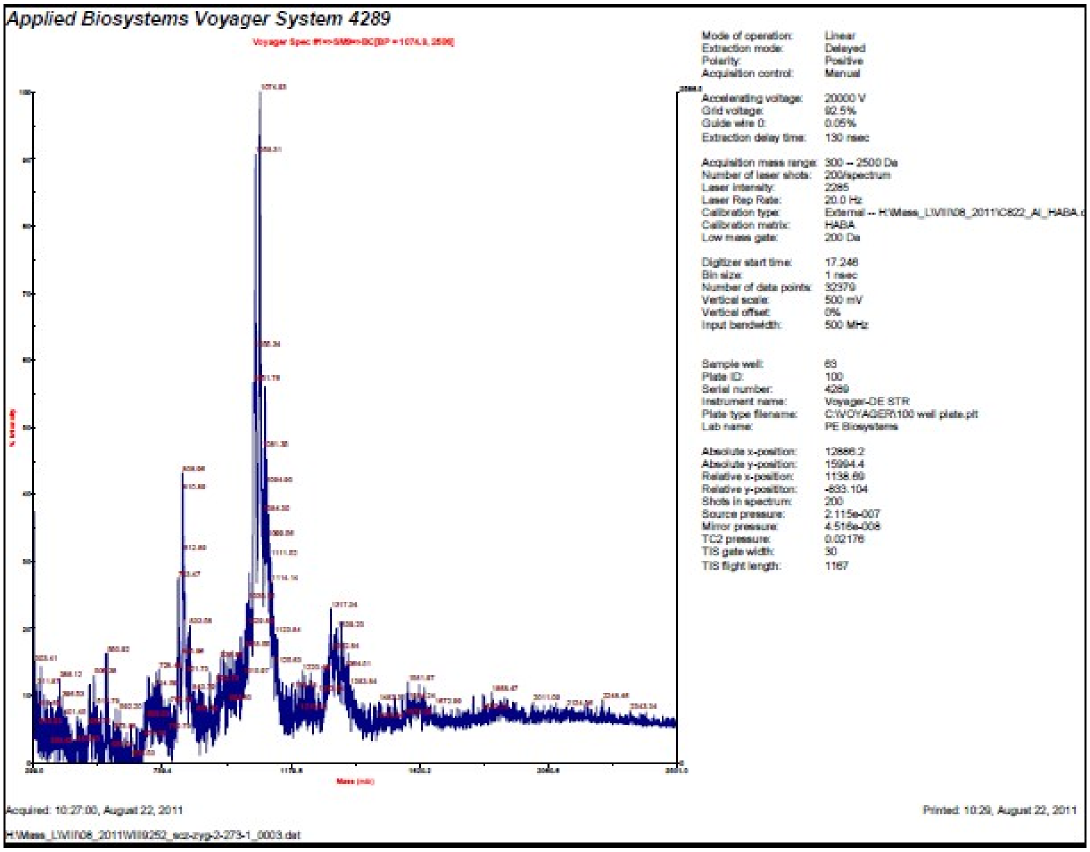

Figure S22. MALDI fragments chart of DeUG-F-monoethoxyl silane monomer modified glass slides surface. Matrix: 2-(4'-hydroxybenzeneazo)benzoic acid (HABA).

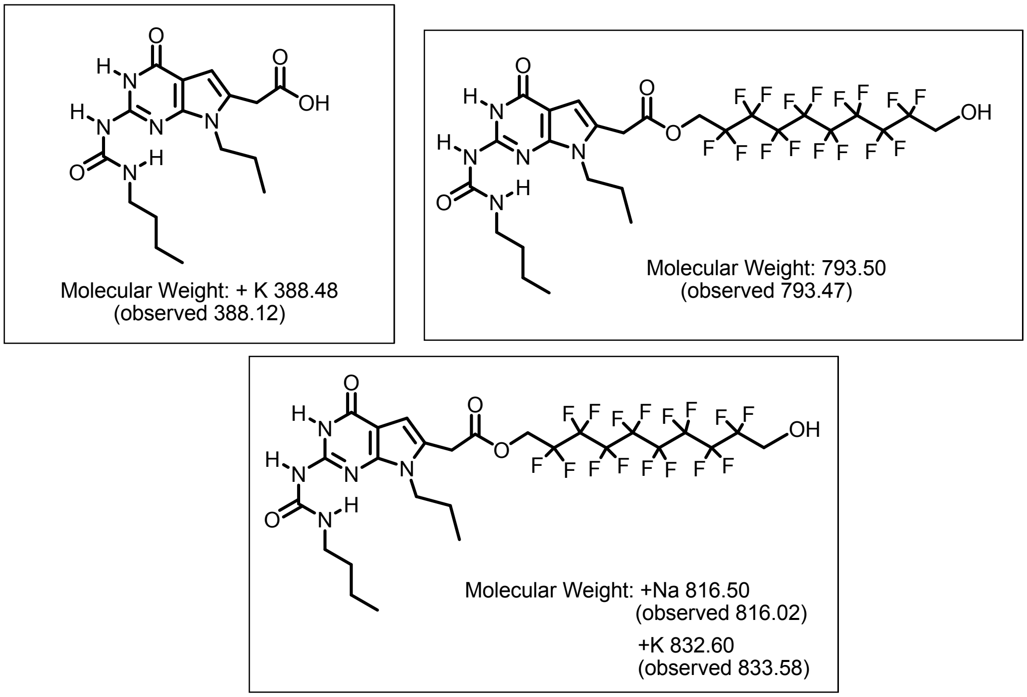

Figure S23. MALDI characteristic fragments of DeUG-F-monoethoxyl silane monomer upon ionizing DeUG-F-monoethoxyl silane monomer modified glass slides surface. Matrix: 2-(4'-hydroxybenzeneazo)benzoic acid (HABA).

6. Thickness of mono-layer on modified Si wafers using ellipsometry measurement

Film thickness of modified silicon wafers was measured using J. A. Woollam Co. variable-angle spectroscopic ellipsometer. Ellipsometric data were acquired via spectroscopic scan with angle of incidence at 50, 60 and 70˚ and spectral range: 300-1000 nm with revolutions per measurement (Revs/Meas) set at 10. Spot sized analyzed was 1 mm in diameter when incident light is normal to the surface, will be larger when scan with angle of incidence at 50, 60 and 70˚.For Piranha treated Si wafer, sequentially add Si, SiO_2 layer, fix Si layer at 1.00 mm, then do a normal fit to obtain thickness of SiO_2 layer (2.23 nm). For surface modified with various silane monomers, sequentially add Si, SiO_2, Cauchy layer, fix Si layer at 1.00 mm, SiO_2 layer at 2.23 nm, then do a normal fit to obtain thickness of SAM layer.

Table S2. Thickness of mono-layer on modified Si wafers using various QHB coupled Silane monomers.

Surface	Calculated thickness (normal to surface) (nm)	Calculated (30˚ with respect to surface normal) (nm)	Measured Thickness (nm)	Mean squared error (MSE)*
Oxidized Silicon wafer			2.23±0.08	1.17
Octyl-tri	1.13	0.97	1.09±0.04	2.22
Octyl-mono	1.13	0.97	0.77±0.02	1.19
Octyl-F-tri	1.13	0.97	1.19±0.02	1.29
Octyl-F-mono	1.13	0.97	1.05±0.02	1.18
UPy-tri	2.38	2.06	2.04±0.07	1.48
UPy-mono	2.38	2.06	1.72±0.05	1.50
(UPy+Octyl)-tri	2.38	2.06	1.93±0.06	1.52
(UPy+Octyl)-mono	2.38	2.06	1.67±0.08	1.53
DeUG-tri	2.13	1.84	1.97±0.06	1.50
DeUG-mono	2.13	1.84	1.78±0.07	1.54
(DeUG+Octyl)-tri	2.13	1.84	1.83±0.06	1.68
(DeUG+Octyl)-mono	2.13	1.84	1.70±0.09	1.56
DAN-tri	2.88	2.49	2.53±0.10	1.50
DAN-mono	2.88	2.49	2.33±0.09	1.57
(DAN+Octyl)-tri	2.88	2.49	2.45±0.11	1.59
(DAN+Octyl)-mono	2.88	2.49	2.27±0.12	1.32
DeUG-F-tri	3.13	2.71	2.85±0.11	1.40
DeUG-F-mono	3.13	2.71	2.71±0.13	1.27
(DeUG-F+Octyl-F)-tri	3.13	2.71	2.77±0.11	1.27
(DeUG-F+Octyl-F)-mono	3.13	2.71	2.67±0.12	1.32

* manual suggests MSE < 10 is a reasonable data fit.

7. Adhesion measurements via Lap-shear experiment

Adhesion was measured using Instron Mini 44 load-frame equipped with a 500 N load-cell and Labview 5.1 software (**Figure S36**). Each lap-shear sample was prepared as following: A pair of glass slides was set using 10 μL of 10 mg/mL of each polymer solution in CH_2Cl_2 with contact area 1.5 cm × 2.5 cm. The sample was clamped with binder clips and cured at room temperature for 24 h before lap shear test. Crosshead speed limit of Instron Mini 44 is 0.05-50 mm/min and maximum load is 50 Kg. For all samples crosshead speed limit was set at 1.0 mm/min. Load (Kg) versus position was plotted and maximum load at fail was recorded. Each data set contains 10 measures. Multiplying the average maximum load at fail by gravitational acceleration constant and divided by contact area give the shear strength in MPa. Error represents plus/minus one standard deviation.

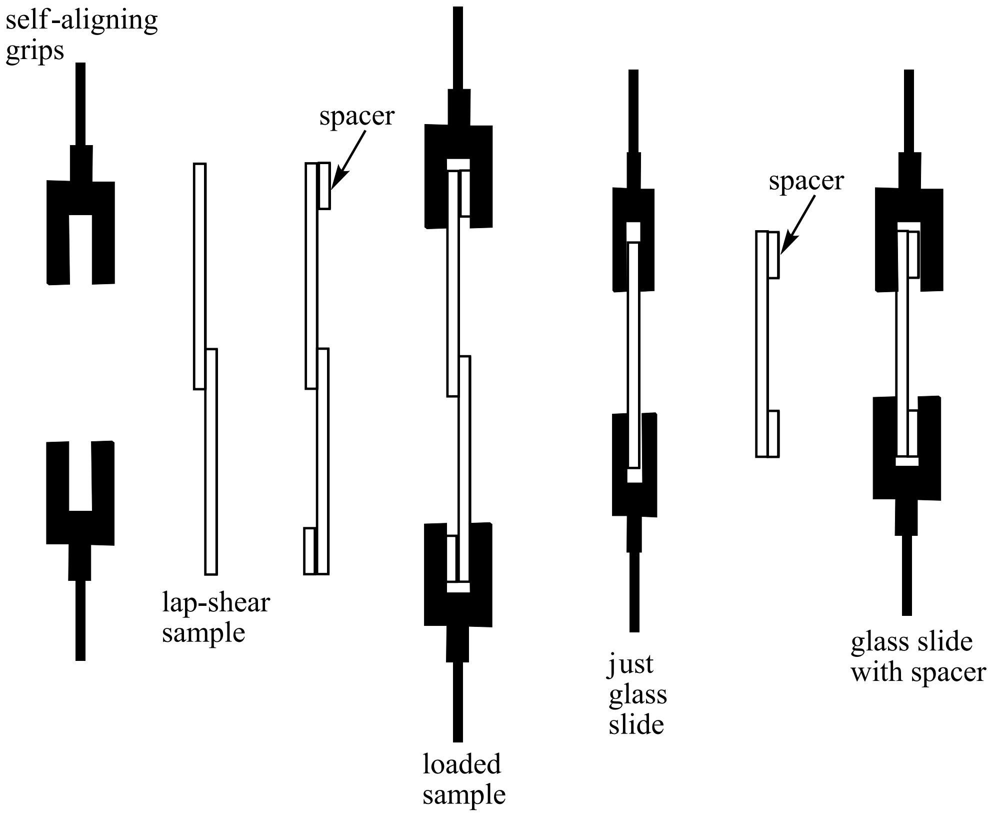

Figure S24. Lap-shear set up, detail of sample loading and controls.

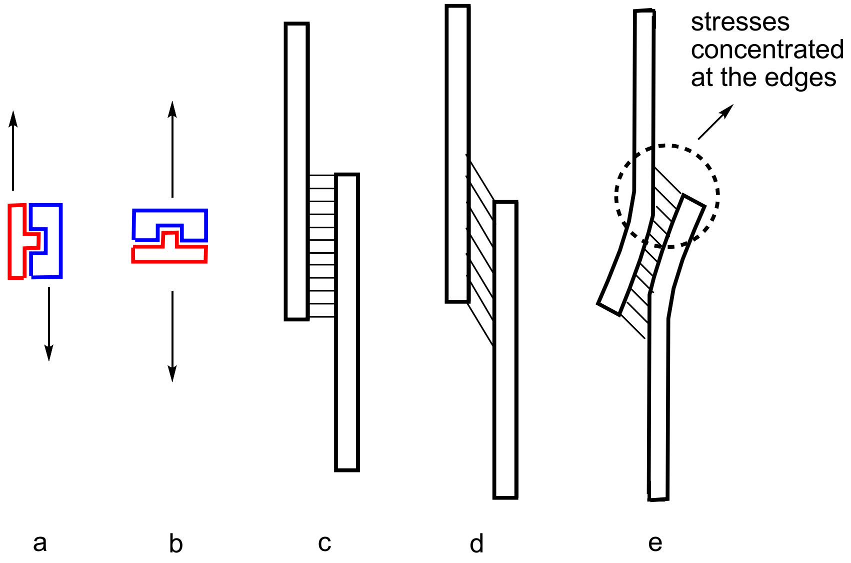

Figure S25. Model of single lap joints using quadruply hydrogen bonding heteo-complex as adhesion promotors. Mechanically rupture quadruply hydrogen bonding heteo-complex (a) perpendicular to H-bonding direction (b) along the H-bonding direction. (c) before loading (d) after loading (e) relief of tearing stress by bending (Volkersen's analysis)[4]

8. Method to calculate single pair rupture force of QHB modules

Gaub *et al* experimentally showed that the unbinding forces of discrete complexes of avidin-biotin are proportional to the enthalpy change of the complex formation but independent of changes in the free energy [5]. Their results indicate that unbinding process is adiabatic and entropic changes occur after unbinding. Molecular mechanics simulation of Streptavidin-Biotin interactions also indicated that rupture strength correlates with enthalpies rather than free energies [6]. Assuming this also applies to disassociation of quadruply hydrogen binding hetero-complex, single pair rupture force of QHB modules can be easily calculated using the method we proposed here.

Firstly, the association/disassociation constant and change of enthalpy (ΔH) need to be measured or derived. Two examples are showed here in detail using Kd and ΔH measured or derived from simple solution chemistry.

9. Theoretical calculation of single pair ruptures force of UPy dimer

For equilibrium of UPy dimer formation:

$$2\ M \underset{Kd}{\overset{Ka}{\rightleftharpoons}} M2$$

$\Delta G = \Delta H - T\Delta S = -RT \ln Ka$

For UPy dimer formation, it is reported [7] that at room temperature 25°C (298.2K) $Ka = 10^7$

Long *et al* [9] showed that UPy dimer can be broken at 80°C (353.2K), assume Ka = 2

Equation can be established as follows:

$\Delta H - T1\Delta S = -RT \ln Ka_1$ $(T1 = 298.2K, Ka_1 = 10^7)$

$\Delta H - T2\Delta S = -RT \ln Ka_2$ $(T2 = 353.2K, Ka_2 = 2)$

$\Delta H - 298.2\Delta S = -8.314*298.2 \ln (10^7)$

$\Delta H - 353.2\Delta S = -8.314*353.2 \ln (2)$

Solve above equations, ΔH = -24559 J/mol (-5.89 Kcal/mol)

In order to break UPy dimer, Mechanical work (energy) is needed:

W=F*S

(W is mechanical work needed to break Hydrogen bonding pair, W can be calculated using ΔH; F is force needed; S is distance of the applied force)

It is known typical hydrogen bonding distance is within 0.1-0.3 nm [8]. It has been showed that for UPy dimer, H-bonding distance is within 0.27-0.32 nm [9]. Estimate pulling apart 0.25 nm breaks UPy dimer, use equation W=F*S single pair rupture force can be calculated as:

$F* (2.5*10^{-10}\ m) = (24559 J/mol)/(6.02*10^{23}/mol)$

Solve equation **F= 160 pN** This calculated single pair rupture force is consistent with the experimental value (**145 pN**) measured by Vancso *et al* [10].

10. Theoretical calculation of single pair rupture force of DeUG-DAN

It was reported that for DeUG-DAN pair [2] in chloroform at 25˚C (298.2K). Ka = $1.9*10^8$, ΔH = -6.9 Kal/mol = -28980 J/mol, TΔS = 2.8 Kal/molCrystal structure showed H-bonding length 0.19-0.20 nm.Estimate pulling apart 0.25 nm breaks DeUG-DAN complex, Use equation W=F*S, single pair rupture force can be calculated as:F* ($2.5*10^{-10}$ m) = (28980J/mol)/($6.02*10^{23}$/mol)

Solve equation **F= 193 pN**

11. Calculation of amount of QHB modules deposited on glass surface

On clean silicon wafers, whose surface is believed to have about 5 $\times 10^{14}$ –SiOH groups/cm^2

The full coverage of perfect SAMs will have 0.83 nmole/cm^2 via silane deposition [11,12] .

Technical data of Goldseal glass Microslides from Ted Pella Inc. showed that silicon dioxide is 72% of its all chemical composition.

Thus the surface coverage can be estimated as 0.83 nmole/cm^2 * 72% = 0.60 nmole/ cm^2

With contact area of lap-shear sample 1.5 cm × 2.5 cm, the amount of QHB modules on each side of glass slides will be 0.6nmol/cm^2*1.5cm*2.5cm=**2.25 nmole**

Each lap-shear sample has two surfaces associated,

So the amount of QHB modules will be **4.50 nmole.**

12. Calculation of amount of QHB modules in PS-DAN, PBMA-DeUG and PBA-UPy.

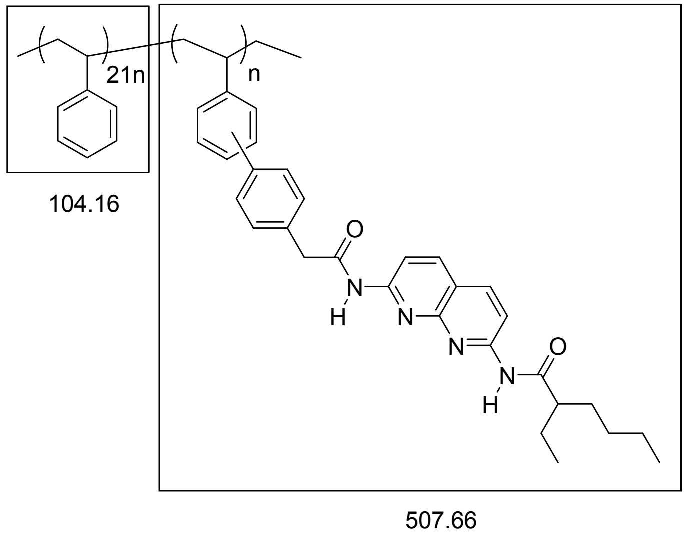

For PS-DAN (4.5 mol %) ***Mn*** **(73 KDa),** 1/22*100% = 4.5 mol %

n*507.66 + 21n*104.16 =73000

solve equation n = 27.1 (n = 27)

In lap-shear, for each pair, use 10 μL (0.01 mL) 10 mg/mL polymer solution

So 0.01 mL*10 mg/mL = 0.1 mg = 0.0001g

(0.0001g/73000g/mol)*27 = 37.0*10^{-9} mol = **37 nmole**

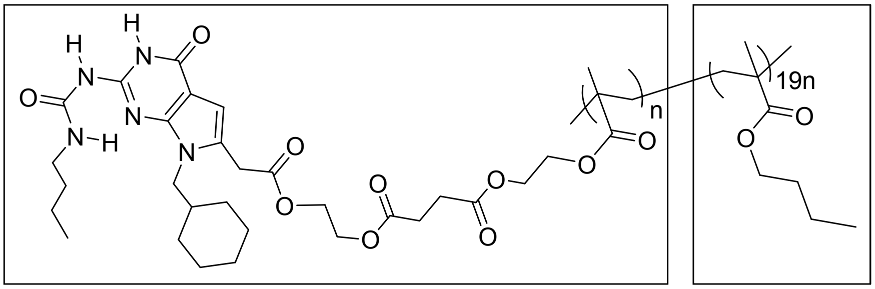

659.73 142.20

For PBMA-DeUG (5.0 mol %) *Mn* (18.5 KDa), 1/20*100% = 5.0 mol %

n*659.73 + 19n*142.20 =18500

solve equation n = 5.5 (n = 6)

In lap-shear, for each pair, use 10 μL (0.01 mL) 10 mg/mL polymer solution

So 0.01 mL*10 mg/mL = 0.1 mg = 0.0001g

(0.0001g/18500g/mol)*6 = 32.4*10^{-9}mol = **32 nmole**

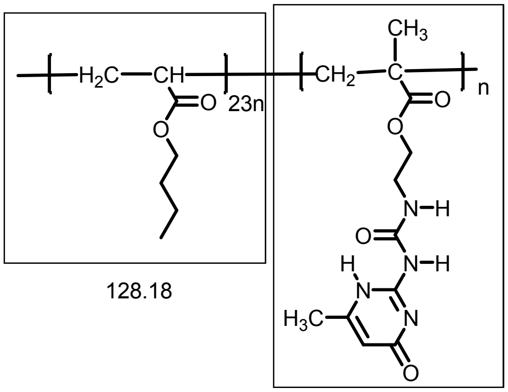

128.18

279.29

For PBA-UPy (4.1 mol %) *Mn* (38 KDa), 1/24*100% = 4.2 mol %

n*279.29 + 23n*128.18 = 38000

solve equation n = 11.8 (n = 12)

In lap-shear, for each pair, use 10 μL (0.01 mL) 10 mg/mL polymer solution

So 0.01 mL*10 mg/mL = 0.1 mg = 0.0001g

(0.0001g/38000g/mol)*12 = 31.6*10^{-9}mol = **32 nmole**

Quantitively there are **32-37 nmole** QHB modules in the amount of polymer used for each lap-shear setting which is 7-8 equivalents to the surface coverage of modified glass surface (0.6 nmol/cm^2, **4.50 nmole** in total for two slides with contact area of 1.5 cm × 2.5 cm). Excessive amount of QHB modules in polymer showed to greatly promote the pairing efficiency, but this will also generate non-specific interactions which should be subtracted when calculating percentage contribution in overall adhesion.

13. Calculation of percentage contribution of QHB modules in overall adhesion

The stress versus extension curve of lap-shear experiment vividly describes the dynamic nature of rupture event. The integration area under the rupture curve was used to calculate the amount of adhesion energy/mechanical work involved in the rupture process. Controls were used to approximate several types of non-specific interactions such as London dispersion and dipole-dipole. Subtracting the energy associated with these non-specific interactions from the overall adhesion, the percentage contribution of specific QHB interaction can be calculated. Adhesion energy/mechanical work was calculated using difference between maximum load at fail and plastic deformation load multiply rupture length, divided by two. Rupture length was defined as difference between position at fail and ending point of plastic deformation of glass slides.

Data set of triethoxyl silane monomers modified surface was chosen for the calculation for the following reasons: (1) it has better surface coverage (2) showed strongest adhesion when using QHB modules modified polymer as adhesion promoters (3) from practical application perspective, it is closest to the of QHB modified polymer systems.

The **apparent rupture length** x of DAN-DeUG pairs can be calculated as following:

$2*0.6*10^{-9}$mole/cm^2*(1.5cm*2.5cm) *6.02*10^{23}*193*10^{-12}N/pair*x = 0.094 J

x = 1.80*10^{-7}m =180 nm =0.180 μm = 0.000180 mm

0.000180 mm = 180 nm is >> 0.25 nm but

0.000180 mm = 180 nm is << 0.92 mm (rupture length/stressed extension in lap-shear)

This implies that stretching the polymer chain and stretching the QHB pair occur at same time over a quite long stretching process.

Overall adhesion using PS-DAN toward DeUG-tri modified glass surface can be calculated. The adhesion here includes specific DAN-DeUG interaction and non-specific interactions.

Length of plateau: 0.17 mm; rupture length: 0.92 mm

Adhesion/mechanical work of the rupture:

(30.0-1.28)Kg*9.806N/Kg*0.00092m*0.5 = 0.130 J

Specific adhesion = overall adhesion-non-specific adhesion

Specific adhesion = overall adhesion-non-(London dispersion 1)-(London dispersion 2)- (dipole-dipole 1)-(dipole-dipole 2)

Specific adhesion = (0.130-0.004-0.003-0.016-0.013) J = 0.094J

0.094/0.130 = 0.723 = 72.3%

72% of adhesion is come from specific DAN-DeUG interaction.

Theoretical adhesion force between two surfaces

Considering the soft nature of H-bonding interaction and those QHB pairs were stained over quite long period of distance (sub mm scale), using apparent rupture length should be a close approximation.

For DAN-DeUG

Use **apparent rupture length** calculated: 180 nm

F_T* (180*10^{-9} m) = ΔH*(2.25*10^{-9} mol)*2= (28980J/mol)*(2.25*10^{-9} mol)*2

Solve equation F_T **= 724.5 N (73.9 Kg)**

Experimental observed average maximum load at fail 29.8 Kg

For UPy-UPy

Use **apparent rupture length** calculated: 180 nm (assuming the same as DAN-DeUG)

F_T* (180*10^{-9} m) = ΔH*(2.25*10^{-9} mol)*2= (24559J/mol)*(2.25*10-9 mol)*2

Solve equation F_T = **614.0 N (62.7 Kg)**

Experimental observed average maximum load at fail 14.2 Kg

14. Calculation of pairing efficiency of QHB modules in Lap-shear

Pairing efficiency for DeUG-DAN complex,

29.8kg/73.9kg* 100% = **40.3 %**

Pairing efficiency for UPy-UPy in lap-shear,

14.2kg/62.7kg* 100% = **22.6 %**

The pairing efficiency of UPy-UPy is about half of that of the DAN-DeUG, which is reasonable considering UPy could also dimerize itself in polymer.

Reference:

(1) Shashoua, V. E.; Sweeny, W.; Tietz, R. F. J. The homopolymerization of monoisocyanates. *J. Am. Chem. Soc*. **1960**, 82, 866-873 .

(2) Kuykendall, D. W.; Anderson, C. A.; Zimmerman, S. C. Hydrogen-bonded DeUG· DAN heterocomplex: structure and stability and a scalable synthesis of DeUG with reactive functionality. *Org. Lett*. **2009**, 11, 61–

64.

(3) Anderson, C. A.; Taylor, P. G.; Zeller, M. A.; Zimmerman, S. C. Room temperature, copper-catalyzed amination of bromonaphthyridines with aqueous ammonia. *J. Org. Chem*. **2010**, 75, 4848–4851.

(4) Wu, S. Polymer Interface and Adhesion. Marcel Dekker. Inc. New York, NY, **1982**, pp 514.

(5) Moy, V. T.; Florin, E. L.; Gaub, H. E. Intermolecular forces and energies between ligands and receptors. *Science*. **1994**, 266, 257-259.

(6) Grubmüller, H.; Heymann, B.; Tavan, P. Ligand binding: molecular mechanics calculation of the streptavidin-biotin rupture force. *Science*. **1996**, 271, 997-999.

(7) Sijbesma, R. P. ; Beijer, F. H.; Brunsveld, L.; Folmer, B. J. B.; Ky Hirschberg, J. H. K.; Lange, R. F. M.; Lowe, J. K. L.; Meijer, E. W. Reversible polymers formed from self-complementary monomers using quadruple hydrogen bonding . *Science*. **1997**, 278, 1601-1604.

(8) Jeffrey, G. A. An Introduction to Hydrogen Bonding. Oxford University Press. New York, NY, **1997**.

(9) Beijer, F. H.; Kooijman, H.; Spek, A. L.; Sijbesma, R. P.; Meijer, E. W. Self-complementarity achieved through quadruple hydrogen bonding . *Angew. Chem. Int. Ed.* **1998**, 37, 75-78.

(10) Zou,S.; Schönherr, H.; Vancso, G. J. Force spectroscopy of quadruple H-bonded dimers by AFM: Dynamic bond rupture and molecular time-temperature superposition. *J. Am. Chem. Soc*., **2005**, 127, 11230-11231.

(11) Wasserman, S. R.; Tao, Y. T.; Whitesides, G. M. Structure and reactivity of alkylsiloxane monolayers formed by reaction of alkyltrichlorosilanes on silicon substrates. *Langmuir*. **1989**, 5, 1074-1087.

(12) Gun, J.; Iseovici, R.; Sagiv, J. On the formation and structure of self-assembling monolayers: II. A comparative study of Langmuir-Blodgett and adsorbed films using ellipsometry and IR reflection-absorption spectroscopy. *J. Colloid Interface Sci*., **1984**, 101, 201-213.
